# Combining Sanford Arylations on Benzodiazepines with the Nuisance Effect

**DOI:** 10.1002/adsc.201700626

**Published:** 2017-08-02

**Authors:** Raysa Khan, Sarote Boonseng, Paul D. Kemmitt, Robert Felix, Simon J. Coles, Graham J. Tizzard, Gareth Williams, Olivia Simmonds, Jessica‐Lily Harvey, John Atack, Hazel Cox, John Spencer

**Affiliations:** ^1^ Department of Chemistry School of Life Sciences University of Sussex Falmer BN1 9QJ UK; ^2^ Oncology AstraZeneca 310 Cambridge Science Park Milton Road Cambridge CB4 0WG UK; ^3^ Tocris Bioscience, the Watkins Building Atlantic Road, Avonmouth Bristol BS11 9QD UK; ^4^ UK National Crystallography Service School of Chemistry University of Southampton Highfield Southampton SO17 1BJ U.K.; ^5^ Sussex Drug Discovery Centre School of Life Sciences University of Sussex Falmer BN1 9QJ UK

**Keywords:** C−H activation, benzodiazepine, photocatalysis, palladacycle, DFT

## Abstract

5‐Phenyl‐1,3‐dihydro‐*2H*‐1,4‐benzodiazepin‐2‐ones react under palladium‐ and visible light photoredox catalysis, in refluxing methanol, with aryldiazonium salts to afford the respective 5‐(2‐arylphenyl) analogues. With 2‐ or 4‐fluorobenzenediazonium derivatives, both fluoroaryl‐ and methoxyaryl‐ products were obtained, the latter resulting from a S_N_Ar on the fluorobenzenediazonium salt (“nuisance effect”). A computational DFT analysis of the palladium‐catalysed and the palladium/ruthenium‐photocalysed mechanism for the functionalization of benzodiazepines indicated that, in the presence of the photocatalyst, the reaction proceeds via a low‐energy SET pathway avoiding the high‐energy oxidative addition step in the palladium‐only catalysed reaction pathway.

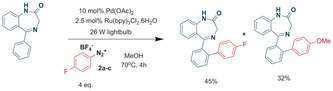

## 1 Introduction

There is a growing impetus for atom economical routes to high value end products employing late stage functionalization (LSF) processes.^1^ These are particularly desirable in medicinal chemistry since they increase diversity and chemical space and enable rapid SAR (structure activity relationship) and ADME‐Tox (Absorption, distribution, metabolism, elimination‐toxicity) feedback that is key to costly, high attrition, drug development. Late stage C−H activation is a powerful tool in generating novel compounds for biological evaluation.^2^ We recently described a palladium‐catalyzed ortho‐arylation of benzodiazepines employing iodonium salts in acetic acid under microwave irradiation.^3^ The harsh conditions, relatively high commercial cost, and multistep synthesis of iodonium salts^4^ (ArIAr’^+^), coupled with a poor atom economy (Ar−I is a byproduct) prompted us to consider a visible‐light photocatalyzed Pd‐mediated protocol involving diazonium salts.^5^

## 2 Results and Discussion

**Scheme 1:**
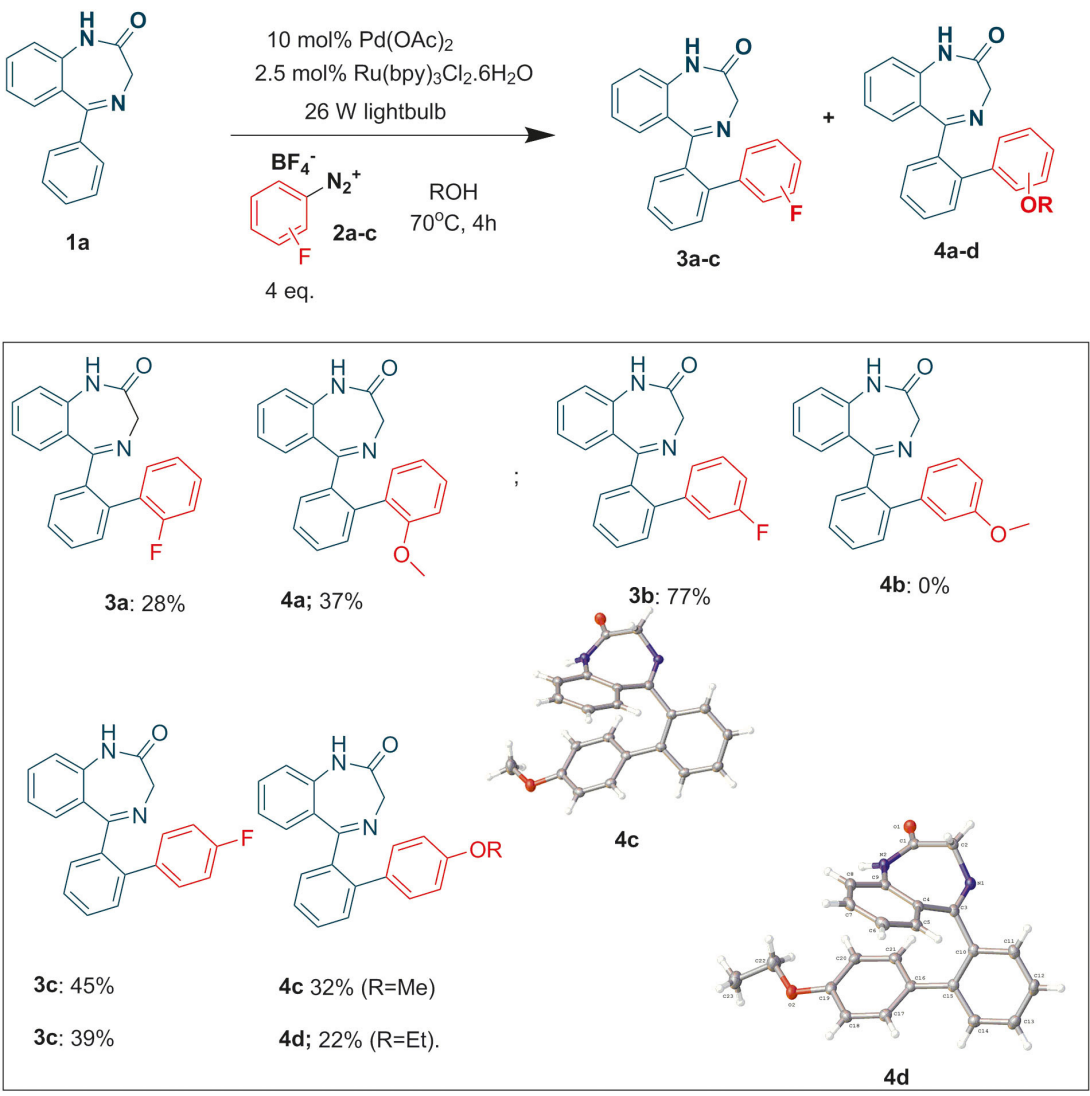
Benzodiazepine library synthesis.

Our initial reaction trials were performed on the benzodiazepine **1 a**, using the 2‐fluoro‐benzenediazonium salt **2 a** under reflux (external oil bath temperature set at 70 °C). To our surprise, in addition to the expected product **3 a**, we were able to isolate the ether product **4 a**. However, reaction of the 3‐isomer **2 b** led exclusively to the fluorobiaryl derivative **3 b**, whereas the 4‐isomer **2 c** afforded a mixture of fluorobiaryl **3 c** and methoxy product **4 c** (Scheme 1). Repeating the reaction in ethanol led to the ethyl ether **4 d** whose x‐ray structure is displayed (Scheme 1).

Characterization of **4 c** was enabled by determination of its solid state x‐ray structure^6^ (Scheme 1) and by its unequivocal synthesis starting from 4‐methoxybenzenediazonium tetrafluoroborate **2 d (**Table 1) where we found slightly better yields under reflux (Entry 1 vs. 2) compared to either ambient temperature or to the absence of photocatalyst (Entry 5). Moreover, a palladium catalyst was essential (Entry 4) for achieving a good yield. Microwave‐mediated chemistry, in the absence of light and photocatalyst, gave little conversion of product.

**Table 1:** Synthesis of an anisole derivative.

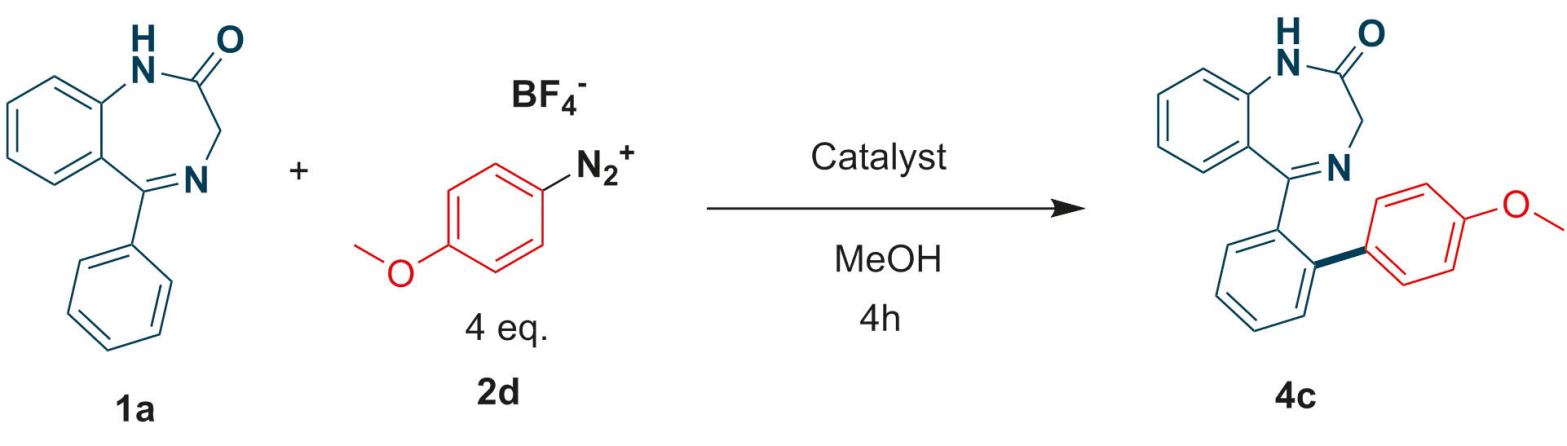					
Entry	Lamps 26 W	Pd(OAc)_2_ (mol%)	Ru(bpy)_3_Cl_2._ 6H_2_O (mol%)	Temp. (^o^C)	Conv. LC/MS (%)
1	Yes	10	2.5	rt	52
2	Yes	10	2.5	Reflux^[a]^	61
3	No	10	2.5	Reflux^[a]^	35
4	Yes	0	2.5	Reflux^[a]^	0
5	Yes	10	0	Reflux^[a]^	57
6	No	10	0	^[b]^	20

^[a]^ External oil bath temperature; 70 °C, ^[b]^ microwave (MW), 125 °C, 1 h.

**Scheme 2:**
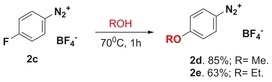
“Nuisance effect” on diazonium salts.

To explain the formation of the ether products we propose a competing S_N_Ar, termed “nuisance effect,” which has historically been observed for halogen‐substituted benzenediazonium salts, given the strong electron withdrawing effects of the diazo group, notably operating on the 2‐ and 4‐substituted isomers.^7^ Indeed, simple alcoholysis of compound **2 c** was achieved in the appropriate alcohol solvent at 70 °C (Scheme 2).

**Scheme 3:**
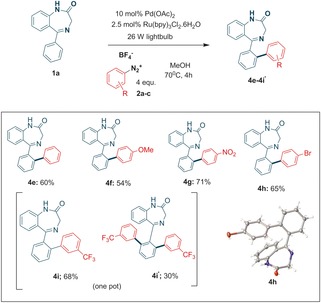
Other arylated benzodiazepines.

The C−H activation reaction was also applied to aryldiazoniums incapable of undergoing such a F‐substitution and, hence derivatives **4 e**–**4 i‘** were synthesized in good to excellent yields (Scheme 3) and the structure of **4h** was determined by x‐ray crystallography. Indeed, yields tend to be either similar or higher than those reported for the corresponding reactions involving iodonium salts, e. g. **4 e** (60% vs. 56%), **4 f** (54% vs. 35%), **4 g** (71% vs. 55%) and **4 i** (64% vs. 63%).

In the synthesis of **4 i**, relatively large amounts of the diarylated adduct **4 i‘** were also observed. Such di‐arylations were previously reported by us.3b

The current and previous library of benzodiazepines (Scheme 1) was tested for GABA binding.^8^ None of the current benzodiazepines displayed any appreciable biological activity although 7‐chloro‐benzodiazepines, as expected, had reasonable activity, although were *ca*. 7–10 fold less active than nordazepam and diazepam controls (Entries 1 and 2 respectively, Table 2) and were not pursued any further.

**Table 2:** GABA activity of library.

Entry	Compound	mean Ki (nM)/ SEM (nM) vs. GABA.
1	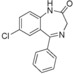	51.62±2.0
2	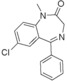	41.41±4.9
3	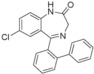	373.45±110.5
4	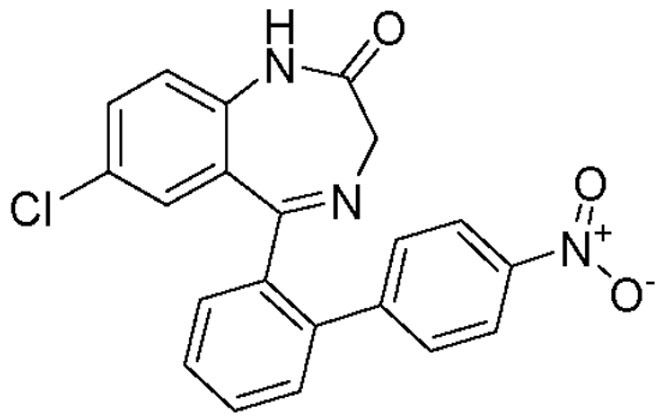	421.54±86.1
5	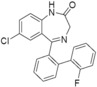	303.25±60.7
6	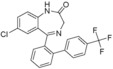	689.56±480.3

**Scheme 4:**
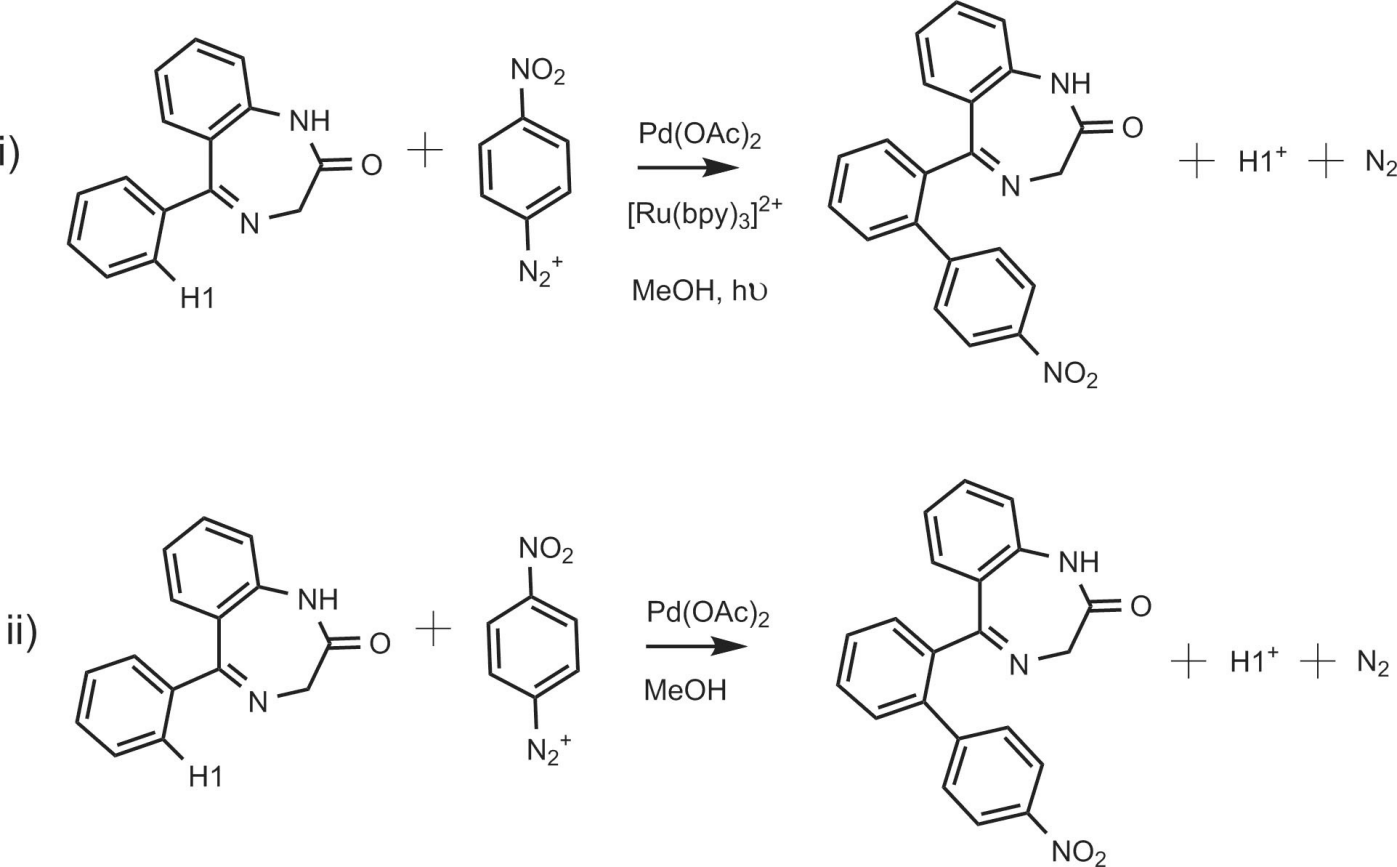
The formation reaction of **4 g** with (i) and without (ii) the Ru photocatalyst, investigated using DFT.

Sanford et al. proposed a possible mechanism to explain their Pd/Ru photocatalysed C−H arylation.5a Here we present a computational study of a Pd‐catalysed and a Sanford‐derived Pd/Ru photocalysed mechanism for the functionalization of **1 a** to **4 g** (Scheme 4) to rationalise the increased yield in the presence of light and a Ru photocatalyst.

**Scheme 5:**
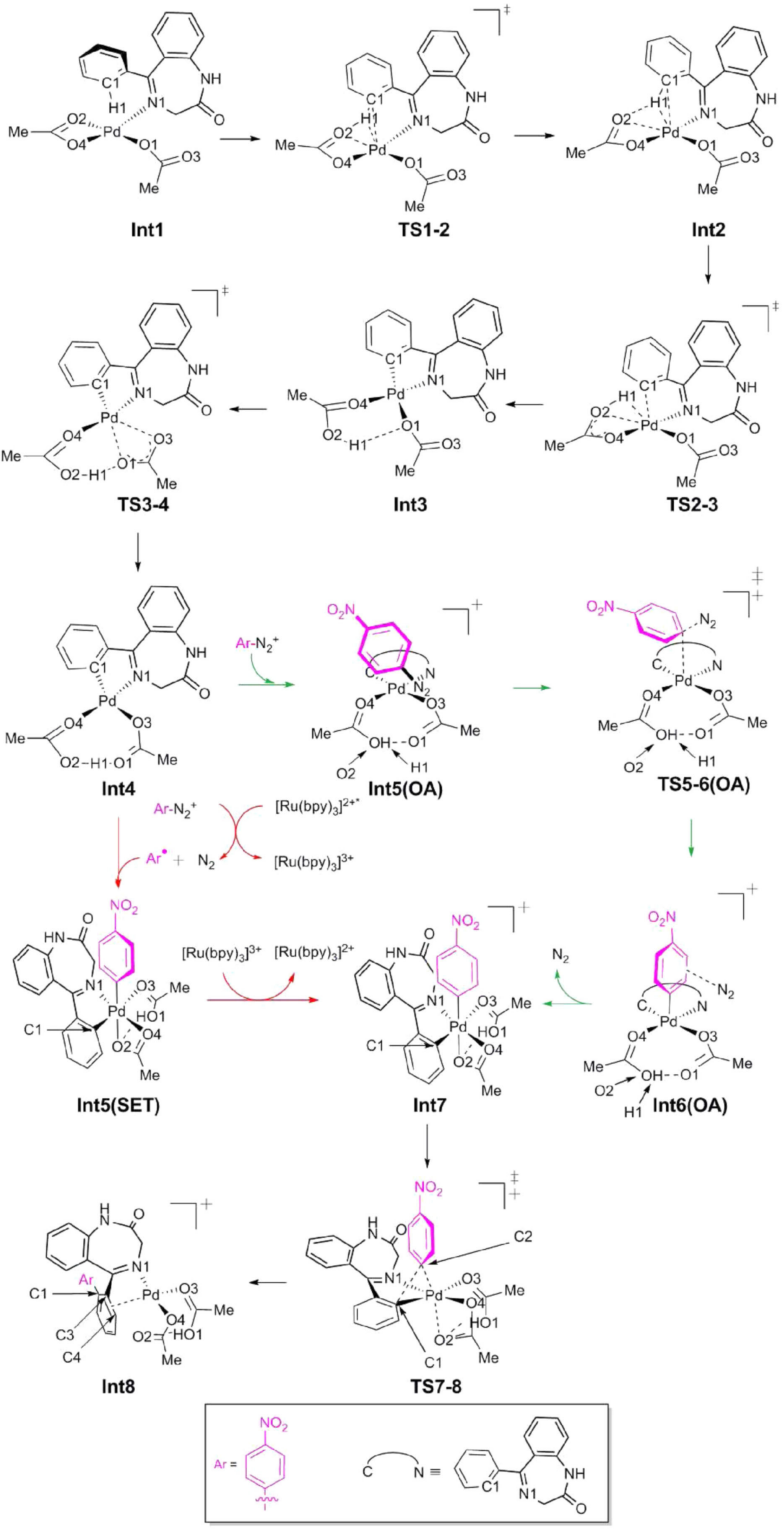
The reaction mechanism for the functionalization of benzodiazepine. From **Int4** to **Int7** the transformation follows the green path in the presence of the Pd catalyst and the red path in the presence of the Pd/Ru catalysts. Both paths were considered.

**Figure 1:**
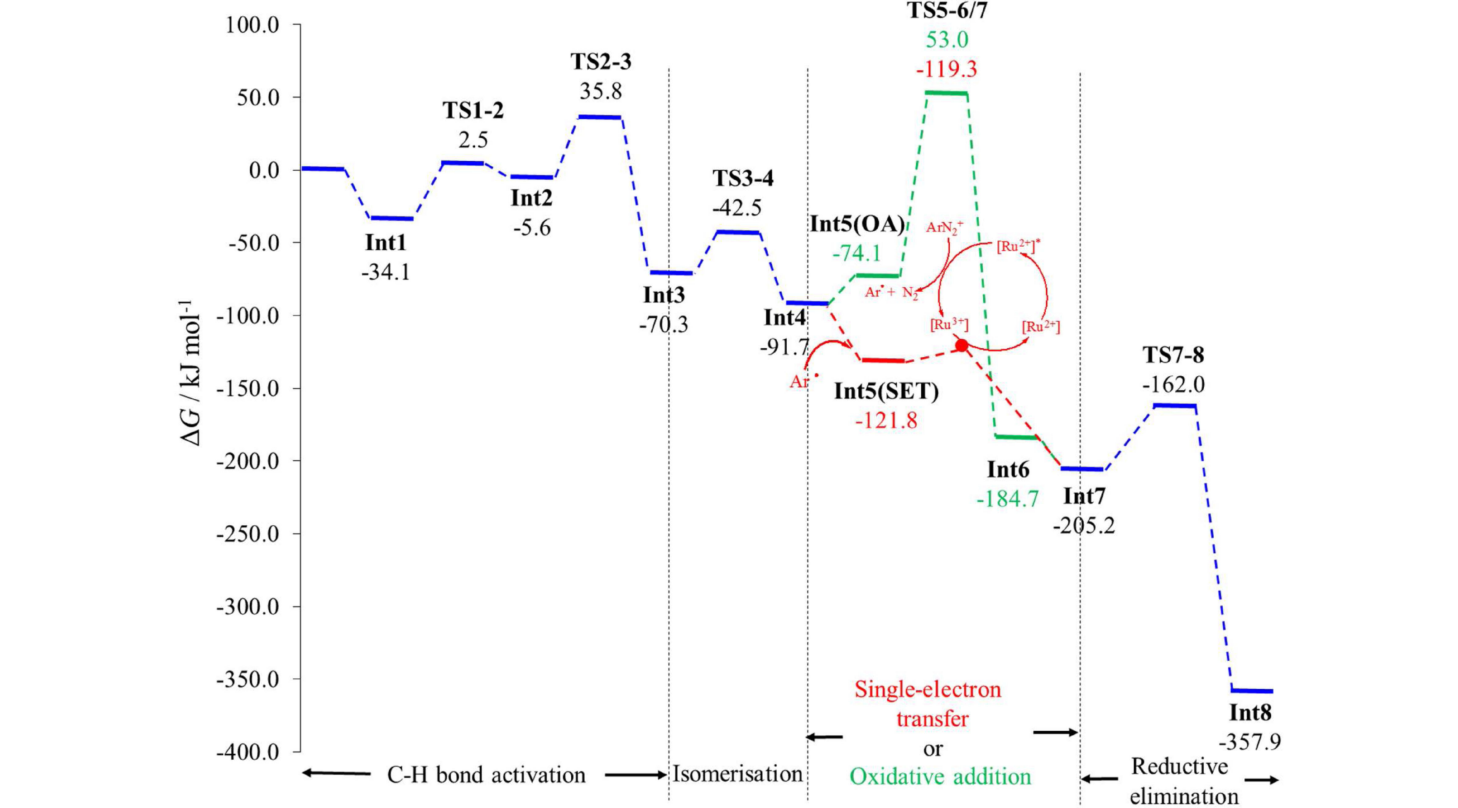
The reaction energy profile for the formation of **4 g** from **1 a**, with (red path) and without (green path) a photocatalyst. Steps common to both mechanisms are shown in blue. [Ru^2+^] and [Ru^3+^] represent [Ru(bpy)_3_]^2+^ and [Ru(bpy)_3_]^3+^, respectively.

The detailed mechanism is shown in Scheme 5 and the reaction profile (relative to the reactants) in Figure 1. The reaction mechanism, with and without the Ru(II)‐photocatalyst, essentially follows the same path except that the oxidative addition step in the presence of just the Pd(II)‐catalyst (path shown in green, Scheme 5 and Figure 1), is replaced by a single‐electron‐transfer (SET) process when the Ru(II)‐photocatalyst is added (shown in red, Scheme 5 and Figure 1).

The initial step of the catalysed mechanism involves the coordination to Pd(OAc)_2_ by a N atom on the un‐functionalised benzodiazepine to provide **Int1**, followed by the formation of an agostic complex **Int2** prior to C−H activation. The atomic distance between Pd and the agostic H in **Int2** is 1.903 Å, which is in good agreement with similar agostic interactions in the literature: Pd−H=1.91 Å^9^ and Rh−H (1.95 Å).^10^ The barrier to C−H bond activation is 41.4 kJ mol^−1^, and involves H migration from C to O *via* a six‐membered ring (**TS2‐3**). Prior to coordination with the *p*‐nitrobenzenediazonium (Ar‐N_2_^+^) the complex undergoes an isomerisation step (**TS3‐4**), which involves a change in the C1−Pd−O3 angle from 132.0 to 172.0 degrees with an energy barrier of 27.8 kJ mol^−1^ to form **Int4**.

In the absence of the photocatalyst, Ar‐N_2_^+^ interacts with the Pd(II) complex and follows an oxidation addition (OA) pathway, (highlighted in green, Scheme 5 and Figure 1). The oxidative addition via **TS5‐6(OA)** has an energy barrier of 127.1 kJ mol^−1^ and involves the formation of an Ar−Pd(IV) complex. The N_2_ is then eliminated leading to **Int7**.

When the Ru(II)‐photocatalyst is present, the nitrobenzene radical (Ar*) is generated from Ar‐N_2_^+^ (via oxidative quenching of Ar‐N_2_^+^ by the photo‐excited [Ru(bpy)_3_]^2+^ complex to form [Ru(bpy)_3_]^3+^)^11^ and follows a single‐electron‐transfer (SET) pathway, (in red, Scheme 5 and Figure 1). The square planar geometry of the Pd(II) complex **Int4** becomes a Pd(III) distorted‐octahedral structure when the Ar binds to the Pd centre in **Int5(SET**); this is consistent with the crystal structure of other Pd(III)‐complexes although we did not consider bimetallic species.^12^ **Int7** is formed directly from **Int5(SET**) by the transfer of an electron to the [Ru(bpy)_3_]^3+^ complex to recover the photocatalyst. The Gibbs free energy barrier for single electron transfer (SET) resulting in the formation of the Pd(IV) complex **Int7** was calculated to be 2.5 kJ mol^−1^ using Marcus and Savéant theory.^13^ The details of this calculation are provided in the Computational Method section. This barrier is very small but similar to literature values that range from 0.4–15.1 kJ mol^−1^.^14^

Both mechanisms (OA and SET) result in the same Pd(IV) structure for **Int7**. At this stage reductive elimination occurs via **TS7‐8** with a barrier of 43.2 kJ mol^−1^. This step involves the formation of a C−C bond to facilitate the functionalization of the benzodiazepine and the oxidation state of the Pd‐center changes from Pd(IV) to Pd(II) (**Int7**→**Int8**). The geometry **Int8**, involves an *η* ^2^(C=C) interaction with Pd. A similar interaction was observed by Ariafard et al.^15^ and Canty et al.^16^ in their DFT calculations and in a palladium complex crystal structure.^17^

It is clear from Figure 1 that, in the presence of the Pd‐catalyst, the oxidative addition step is rate determining with a considerable energy barrier. However, in the presence of both the Pd(II)‐catalyst and the Ru(II)‐photocatalyst this OA step, and hence large energy barrier, is avoided as the reaction proceeds via a very low‐energy single‐electron‐transfer process. This provides a rationale for the increased yield in the presence of a photocatalyst.

## Conclusion

The C−H activation of benzodiazepines with 2‐ or 4‐fluorobenzene diazonium salts under Pd catalysis with a Ru photocatalyst, in alcohol solvent, under reflux, leads to a mixture of both fluoroaryl and alkoxyaryl products. Reaction temperature is a key factor in determining the ratio of expected vs. “nuisance effect” (S_N_Ar) products. At ambient temperature trace amounts of the S_N_Ar product are detected whereas significant amounts can be obtained after prolonged heating under reflux. This process can also be extended to other aryl diazonium salts affording ortho‐arylated benzodiazepines. These were tested for biological activity but were found to be significantly less active than e. g. nordazepam and diazepam controls. Density functional theory (DFT) has been used to provide a detailed mechanistic understanding of the functionalization of the benzodiazepines and to offer an explanation for the increased yield in the presence of a Ru(II)‐photocatalyst. The Pd/Ru catalytic cycle follows the mechanism proposed by Sandford et al.5a The increased yield in the visible‐light photocatalysed Pd‐mediated protocol is attributed to the transformation step leading to the formation of the Pd(IV) complex. In the presence of the photocatalyst the reaction proceeds via a low‐energy SET pathway and avoids the high‐energy oxidative addition step in the Pd‐only catalysed reaction pathway.

Current studies are aiming to extend the arylation/nuisance effect chemistry to a wider scope of privileged structures with different nucleophiles for application in medicinal chemistry library generation and will be reported in due course.

## **Experimental Section**

### General Information

All reactions were conducted under an inert atmosphere unless specified otherwise. All commercially purchased materials and solvents were used without further purification unless specified otherwise.

NMR spectra were recorded on a Varian V NMRS 500 (^1^H: 500 MHz, ^13^C: 126 MHz) spectrometer and prepared in deuterated solvents such as CDCl_3_ and DMSO‐d_6_. ^1^H and ^13^C chemical shifts were recorded in parts per million (ppm). Multiplicity of ^1^H‐NMR peaks are indicated by s – singlet, d – doublet, dd – doublets of doublets, t – triplet, pt – pseudo triplet, q – quartet, m – multiplet and coupling constants are given in Hertz (Hz).

Electrospray ionisation – high resolution mass spectra (ESI‐HRMS) were obtained using a Bruker Daltonics Apex III where Apollo ESI was used as the ESI source. All analyses were conducted by Dr A. K. Abdul‐Sada at Sussex. The molecular ion peaks [M]+ were recorded as mass to charge (m/z) ratio.

LC–MS spectra were acquired using a Shimadzu LC–MS 2020, on a Gemini 5 μm C18 110 Å column and percentage purities were run over 30 minutes in water/acetonitrile with 0.1% formic acid (5 min at 5%, 5%–95% over 20 min, 5 min at 95%) with the UV detector at 254 nm. Purifications were performed by flash chromatography on silica gel columns or C18 columns using a Combi flash RF 75 PSI, ISCO unit. The following CCDC deposition numbers have been obtained, in parentheses; for **4 c** (1518056), **4 d** (1551609) and **4 h** (1551610).

### 1 ‐Methoxybenzenediazonium Tetrafluoroborate (2 d)

A stirred suspension of 4‐fluorobenzenediazonium tetrafluoroborate (0.10 g, 0.48 mmol) in methanol (2 mL) was heated at 70 °C by using an external oil bath for 1 hour. The reaction was allowed to cool to ambient temperature and concentrated under reduced pressure. The residue was precipitated by the addition of diethyl ether and collected by filtration, affording **2 d** as a white solid (0.090 g, 85%). The spectral data were concurrent with those reported.^18^

### 2 ‐Ethoxybenzenediazonium Tetrafluoroborate (2 e)

The reaction was conducted by the same procedure as for **2 d** but ethanol (2 mL) was used instead of methanol and heated at 70 °C for 1 hour. **2 e** was obtained as a white solid (0.071 g, 63%). The spectral data were concurrent with those reported.^19^

### 3 ‐Methoxybenzenediazonium Tetrafluoroborate (2 f)

The reaction was conducted by the same procedure as for **2 d** but 2‐fluorobenzenediazonium tetrafluoroborate (0.10, 0.48 mmol) was used instead. **2 f** was obtained as a white solid (0.073 g, 72%). The spectral data were concurrent with those reported.

### 4 ‐(2’‐Fluorobiphenyl‐2‐yl)‐1,3‐dihydro‐2H‐1,4‐benzodiazepin‐2‐one (3 a); 5‐(2’‐methoxybiphenyl‐2‐yl)‐1,3‐dihydro‐2H‐1,4‐benzodiazepin‐2‐one (4 a)

5‐Phenyl‐1,3‐dihydro‐2H‐1,4‐benzodiazepin‐2‐one (0.070 g, 0.3 mmol), 2‐fluorobenzenediazonium tetrafluoroborate (0.25 g, 1.20 mmol) and palladium (II) acetate (0.0067 g, 0.03 mmol) were suspended in degassed, anhydrous methanol (5 mL). Two fluorescent light bulbs (26 W) were placed on either side of the reaction vessel and the reaction mixture was heated at 70 °C by using an external oil bath for 4 hours. The reaction was allowed to cool to ambient temperature, diluted with ethyl acetate (50 mL), washed with water (20 mL) and aqueous sodium sulphite (10%, 35 mL ×2). The layers were separated and the combined aqueous layers were extracted with ethyl acetate (50 mL). Thereafter the combined organic layer was washed with brine (50 mL), dried (MgSO_4_) and concentrated under reduced pressure. The resulting crude material was purified by reversed phase chromatography (water/acetonitrile with 0.1% formic acid, 5 min at 0%, 30%–90%). Starting material **1 a** was recovered (0.014 g, 0.06 mmol). Two products were generated; **3 a** was obtained as a white solid (0.022 g, 28%) and **4 a** was obtained as a white solid (0.030 g, 37%). **3 a**: The spectral data were concurrent with those reported.^3^ **4 a**: ^1^H‐NMR (500 MHz) CDCl_3_: δ=7.98 (s, N**H**, 1H), 7.68 (d, ^3^ *J_HH_*=7.0 Hz, Ar**H**, 1H), 7.52–7.42 (m, Ar**H**, 2H), 7.28 (d, ^3^ *J_HH_*=8.0 Hz, Ar**H**, 1H), 7.19–7.12 (m, Ar**H**, 1H), 7.06–6.98 (m, Ar**H**, 2H), 6.90–6.83 (m, Ar**H**, 1H), 6.80 (d, ^3^ *J_HH_*=7.5 Hz, Ar**H**, 1H), 6.69–6.60 (m, Ar**H**, 2H), 6.52 (d, ^3^ *J_HH_*=8.0 Hz, Ar**H**, 1H), 4.22 (s, COC**H_2_**, 2H), 3.51 (s, O‐C**H_3_**, 3H). ^13^C‐NMR (126 MHz) CDCl_3_: δ=173.1(**C**=O), 171.1 (**C**=N), 156.1 (Ar**C**), 140.8 (Ar**C**), 139.0 (Ar**C**), 137.4 (Ar**C**), 131.3 (Ar**C**), 131.5 (Ar**C**), 131.4 (Ar**C**), 131.1 (Ar**C**), 130.3 (Ar**C**), 129.8 (Ar**C**), 129.6 (Ar**C**), 129.3 (Ar**C**), 128.9 (Ar**C**), 127.7 (Ar**C**), 123.3 (Ar**C**), 120.3 (Ar**C**), 120.2 (Ar**C**), 110.0 (Ar**C**), 56.7 (CO**C**H_2_), 55.3 (O‐**C**H_3_). HRMS‐ESI (m/z) calculated for C_22_H_18_FN_2_O_2_ [+H] ^+^: 343.1441, found: 343.1446. LCMS purity (UV)=96%, tR 10.63 min.

### 5 ‐(3’‐Fluorobiphenyl‐2‐yl)‐1,3‐dihydro‐2H‐1,4‐benzodiazepin‐2‐one (3 b)

The reaction was conducted on a 0.20 mmol scale by the same procedure as for **3 a**/**4 a** but 3‐fluorobenzenediazonium tetrafluoroborate (0.17 g, 0.8 mmol) was used instead of 2‐fluorobenzenediazonium tetrafluoroborate. Starting material, **1 a** was recovered (0.010 g, 0.042 mmol) and **3 b** was obtained as a white solid (0.040 g, 77%). The spectral data were concurrent with those reported.

### 6 ‐(4’‐Fluorobiphenyl‐2‐yl)‐1,3‐dihydro‐2H‐1,4‐benzodiazepin‐2‐one (3 c); 5‐(4’‐Methoxybiphenyl‐2‐yl)‐1,3‐dihydro‐2H‐1,4‐benzodiazepin‐2‐one (4 c)

This reaction was conducted on a 0.42 mmol scale by the same procedure as **3 a**/**4 a** and 4‐fluorobenzenediazonium tetrafluoroborate (0.35 g, 1.67 mmol) was used instead of 2‐fluorobenzenediazonium tetrafluoroborate. Starting material, **1 a** was recovered (0.015 g, 0.06 mmol) and the reaction generated two products; **3 c** was obtained as a white solid (0.053 g, 45%) and **4 c** was obtained as a white solid (0.038 g, 32%). **3 c**: ^1^H‐NMR (500 MHz) DMSO‐d_6_: δ=10.39 (s, Ar**H**, 1H), 7.60–7.55 (m, 1H), 7.55–7.52 (m, Ar**H**, 1H), 7.50 (d, ^3^ *J_HH_*=7.5, Hz, Ar**H**, 1H), 7.33–7.30 (m, Ar**H**, 1H), 7.21–7.17 (m, Ar**H**, 1H), 6.92–6.86 (m, Ar**H**, 4H), 6.83–6.77 (m, Ar**H**, 2H), 6.69 (d, ^3^ *J_HH_*=8.0 Hz, 1H), 4.03 (s, COC**H_2_**, 2H).

^13^C‐NMR (126 MHz) DMSO‐D_6_: δ=172.1 (**C**=O), 169.7 (**C**=N), 161.5 (d, ^1^ *J*_FC_=244.0 Hz, Ar**C**,), 140.4 (Ar**C**), 139.8 (Ar**C**), 139.2 (Ar**C**), 136.9 (Ar**C**), 131.5 (Ar**C**), 130.4 (A r**C**), 130.5 (d, ^3^ *J*_FC_=7.5 Hz, 2× Ar**C**), 130.2 (Ar**C**), 130.1 (Ar**C**), 129.3 (Ar**C**), 128.3 (Ar**C**), 127.8 (Ar**C**), 122.7 (Ar**C**), 120.7 (Ar**C**), 114.9 (d, ^2^ *J*_FC_=22.0 Hz, 2 x Ar**C**), 57.3 (CO**C**H_2_). HRMS‐ESI (m/z) calculated for C_21_H_15_FN_2_O [+H]^+^: 331.1241, found: 331.1244. LCMS purity (UV)=92%, tR 11.16 min. **4 c**: The spectral data were concurrent with those reported.

### 7 ‐(4’‐Ethoxybiphenyl‐2‐yl)‐1,3‐dihydro‐2H‐1,4‐benzodiazepin‐2‐one (4 d)

The same method as that of **3 a**/**4 a** was used but ethanol (5 mL) was used as the solvent instead of methanol. Starting material, **1 a**, was recovered (0.020 g, 0.085 mmol). Two products were generated, product **3 c** was obtained as a white solid (0.043 g, 39%) and Product **4 d** was obtained as a white solid (0.026 g, 22%). **4 d**: ^1^H‐NMR (500 MHz) CDCl_3_: δ=8.20 (s, N**H,** 1H), 7.68 (d, ^3^ *J_HH_*=7.5 Hz, Ar**H**, 1H), 7.57–7.38 (m, Ar**H**, 2H), 7.28 (d, ^3^ *J_HH_*=7.5 Hz, Ar**H**, 1H), 7.15 (pt, ^3^ *J_HH_*=7.5 Hz, Ar**H**, 1H), 6.91–6.81 (m, Ar**H**, 4H), 6.69 (d, ^3^ *J_HH_*=8.0 Hz, Ar**H**, 1H), 6.60 (d, ^3^ *J_HH_*=8.0 Hz, Ar**H**, 2H), 4.29 (s, COC**H_2_**, 2H), 3.94 (q, ^3^ *J_HH_*=7.0 Hz, O‐C**H_2_**CH_3_, 2H), 1.36 (t, ^3^ *J_HH_*=7.0 Hz, O‐CH_2_C**H_3_**, 3H). ^13^C‐NMR (126 MHz) CDCl_3_: δ=173.2 (**C**=O), 170.7(**C**=N), 157.8 (Ar**C**), 141.7 (Ar**C**), 139.5 (Ar**C**), 137.3 (Ar**C**), 133.2 (Ar**C**), 131.1 (Ar**C**), 130.1 (Ar**C**), 129.9 (Ar**C**), 129.7 (Ar**C**), 129.8 (2× Ar**C**), 129.5 (Ar**C**), 129.1 (Ar**C**), 128.1 (Ar**C**), 126.9 (Ar**C**), 123.1 (Ar**C**), 113.8 (2 x Ar**C**), 63.5 (O‐**C**H_2_CH_3_), 56.5 (CO**C**H_2)_, 14.8 (O‐CH_2_ **C**H_3_). HRMS‐ESI (m/z) calculated for C_23_H_20_N_2_O_2_ [+Na] ^+^: 379.1417, found: 379.1419. LCMS purity (UV)=87%, tR 10.89 min.

### 8 ‐Phenyl‐2‐yl)‐1,3‐dihydro‐2H‐1,4‐benzodiazepin‐2‐one (4 e)

The reaction was conducted by the same procedure as for **3 a**/**4 a** but benzenediazonium tetrafluoroborate (0.23 g, 1.20 mmol) was used instead of 2‐fluorobenzenediazonium tetrafluoroborate. Starting material **1 a** was recovered (0.016 g, 0.067 mmol) and **4 e** was obtained as a white solid (0.043 g, 60%). All spectral data were concurrent with those reported.

### 9 ‐(4’‐Methoxybiphenyl‐2‐yl)‐1,3‐dihydro‐2H‐1,4‐benzodiazepin‐2‐one (4 f)

The reaction was conducted on a 0.32 mmol scale by the same procedure as for **3 a**/**4 a** but 4‐methoxybenzenediazonium tetrafluoroborate (0.28 g, 1.28 mmol) was used instead of 2‐fluorobenzenediazonium tetrafluoroborate. Starting material, **1 a** was recovered (0.015 g, 0.063 mmol) and **4 f** was obtained as a white solid (0.048 g, 54%). All spectral data were concurrent with those reported.

### 10 ‐(4’‐Nitrobiphenyl‐2‐yl)‐1,3‐dihydro‐2H‐1,4‐benzodiazepin‐2‐one (4 g)

The reaction was conducted on a 0.45 mmol scale by the same procedure as for **3 a**/**4 a** but 4‐nitrobenzenediazonium tetrafluoroborate (0.43 g, 1.80 mmol) was used instead. Starting material, **1 a** was recovered (0.020 g, 0.085 mmol) and **4 g** was obtained as a white solid (0.093 g, 71%). ^1^H‐NMR (500 MHz) CDCl_3_: δ=8.78 (s, 1H), 7.94 (dd, ^3^ *J_HH_*=8.5, 1.5 Hz, 2H), 7.78–7.72 (m, 1H), 7.60–7.52 (m, 2H), 7.34–7.28 (m, 1H), 7.22–7.13 (m, 3H), 6.90–6.83 (m, 2H), 6.72 (d, ^3^ *J_HH_*=8.0 Hz, 1H), 4.31 (s, COC**H_2,_** 2H). ). ^13^C‐NMR (126 MHz) CDCl_3_: δ=172.1 (**C**=O), 170.6 (**C**=N), 147.4 (Ar**C**), 146.6 (Ar**C**), 139.7 (Ar**C**), 139.6 (Ar**C**), 137.5 (Ar**C**), 131.7 (Ar**C**), 130.4 (Ar**C**), 129.9 (Ar**C**), 129.8 (Ar**C**), 129.6 (Ar**C**), 129.5 (Ar**C** x 2), 128.7 (Ar**C**), 128.6 (Ar**C**), 123.3 (Ar**C**), 122.7 (Ar**C** x 2), 120.1 (Ar**C**), 56.5 (CO**C**H_2_). HRMS‐ESI (m/z) calculated for C_21_H_15_N_3_O_3_ [+H] ^+^: 358.1186, found: 358.1191. Elemental Analysis: Calculated for C_21_H_15_N_3_O_3_ (%): C, 70.58, H, 4.23, N, 11.76, found: C, 70.41, H, 4.23, N, 11.60.

### 11 ‐(4’‐Bromobiphenyl‐2‐yl)‐1,3‐dihydro‐2H‐1,4‐benzodiazepin‐2‐one (4 h)

The reaction was conducted on a 0.25 mmol scale by the same procedure as for **3 a**/**4 a** but 4‐bromobenzenediazonium tetrafluoroborate (0.27 g, 1.0 mmol) was used instead. Starting material, **1 a** was recovered (0.012 g, 0.051 mmol) and **4 h** was obtained as a white solid (0.051 g, 65%). ^1^H‐NMR (500 MHz) DMSO‐d_6_: δ=10.44 (s, 1H), 7.56 (pt, ^3^ *J_HH_*=8.0 Hz, 2H), 7.54–7.48 (m, 1H), 7.34 (d, ^3^ *J_HH_*=8.0 Hz, 1H), 7.28 (d, ^3^ *J_HH_*=8.0 Hz, 2H), 7.23 (pt, ^3^ *J_HH_*=7.5 Hz, 1H), 6.87 (dd, *J*=8.0, 5.9 Hz, 3H), 6.82 (pt, ^3^ *J_HH_*=7.5 Hz, 1H), 6.73 (d, ^3^ *J_HH_*=8.0 Hz, 1H), 4.05 (s, COC**H_2_**, 2H). ^13^C‐NMR (126 MHz) DMSO‐d_6_: δ=171.9 (**C**=O), 169.8 (**C**=N), 140.2 (Ar**C**), 139.8 (Ar**C**), 139.6 (Ar**C**), 139.3 (Ar**C**), 131.5 (Ar**C**), 131.1 (Ar**C**), 131.0 (Ar**C** ×2), 130.6 (Ar**C ×**2), 130.3 (Ar**C**), 130.2 (Ar**C**), 130.1 (Ar**C**), 129.5 (Ar**C**), 128.2 (Ar**C**), 128.1 (Ar**C**), 122.7 (Ar**C**), 120.7 (Ar**C**), 56.7 (CO**C**H_2_). HRMS‐ESI (m/z) calculated for C_21_H_15_BrN_2_O [+H] ^+^: 391.0441, found: 391.0449. LCMS purity (UV)=95%, tR 14.56 min.

### 12 ‐(3’‐Trifluoromethylbiphenyl‐2‐yl)‐1,3‐dihydro‐2H‐1,4‐benzodiazepin‐2‐one (4 i); 5‐(3,3’‐bistrifluoromethylbiphenyl‐2,6‐yl)‐1,3‐dihydro‐2H‐1,4‐benzodiazepin‐2‐one (4 i‘)

The reaction was conducted on a 0.39 mmol scale by the same procedure as for **3 a**/**4 a** but 3‐trifluoromethylbenzenediazonium tetrafluoroborate (0.41 g, 1.56 mmol) was used instead. **4 i** was obtained as a brown solid (0.094 g, 64%) and the bisarylated product, **4 i‘**, was obtained as a brown solid (0.061 g, 30%). **4 i**: All spectral data were concurrent with those reported. **4 i‘**: ^1^H‐NMR (500 MHz) DMSO‐d_6_: δ=10.01 (s, 1H), 7.66 (pt, ^3^ *J_HH_*=7.5 Hz, 1H), 7.55 (d, ^3^ *J_HH_*=8.0 Hz, 2H), 7.50 (d, ^3^ *J_HH_*=7.5 Hz, 2H), 7.45 (pt, ^3^ *J_HH_*=7.5 Hz, 2H), 7.41–7.36 (m, 4H), 7.22–7.18 (m, 1H), 6.96–6.89 (m, 2H), 6.75 (d, *J*=8.0 Hz, 1H), 3.65 (s, COC**H_2_**, 2H). ^13^C‐NMR (126 MHz) CDCl_3_: δ=170.4 (**C**=O), 169.6 (**C**=N), 141.4 (Ar**C**), 141.0 (Ar**C**), 138.1 (Ar**C**), 137.4 (Ar**C**), 132.4 (Ar**C ×**2), 131.7 (Ar**C**), 130.1 (q, ^2^ *J*_FC_, 33 Hz, Ar**C** ×2), 129.8 (Ar**C** ×2), 129.4 (Ar**C ×**2), 129.3 (Ar**C ×**2), 128.7 (Ar**C**), 128.2 (Ar**C ×**2), 125.8 (q, ^3^ *J*_FC_, 3.5 Hz, Ar**C ×**2), 123.9 (q, ^3^ *J*_FC_, 272.0 Hz Ar**C ×**2), 123.7 (q, ^3^ *J*_FC_, 3.5 Hz, Ar**C** x 2), 123.4 (Ar**C**), 120.2 (Ar**C**), 55.7 (CO**C**H_2_). C_29_H_18_F_6_NO_2_ [+H] ^+^: 525.1396, found: 525.1402. LCMS purity (UV)=98%, tR 22.50 min.

## 3 Computational Details

Density functional theory (DFT) calculations were performed at the ωB97XD/6‐311++G(2df,2p)[SDD]//PBE/6‐31+G(d,p)[SDD] level of theory, using the Gaussian09 program.^20^ The Pople basis sets were used on all atoms except Pd and Ru for which the SDD relativistic effective core potentials were used.^21^ The PBE functional^22^ was used for the geometry optimisation and frequency analysis as it combines good accuracy for Pd complexes with computational speed.^23^ The long‐range corrected hybrid functional ωB97XD,^24^ which includes empirical dispersion corrections, was used for energies to ensure accurate energetics.^25^ Methanol solvent energy corrections were applied using the conductor‐like polarisable continuum model (CPCM).^26^ Accordingly, the Gibbs free energies presented in Figure 1 were obtained by adding the thermal free energy corrections obtained at the PBE/6‐31+G(d,p)[SDD] level of theory to the solvent‐corrected electronic energies obtained at the ωB97XD/6‐311++G(2df,2p)[SDD] level of theory. All stationary states were verified as minima or transition states by the absence or presence, respectively, of a single imaginary vibrational frequency. Eigenvector following was used to ensure transition states connected the desired minima.

The Gibbs free energy barrier for single electron transfer (SET), $\Delta G_{ET}^{\neq }$ , was calculated using the following equation from Marcus and Savéant theory:13b–13d

(1) $$\Delta G_{ET}^{\neq }=\Delta G_{0}^{\neq }{\left[1+\frac{\Delta G_{r}}{4\Delta G_{0}^{\neq }}\right]}^{2}$$

Here $\Delta G_{r}$ is the reaction energy for the electron transfer step and $\Delta G_{0}^{\neq }$ is the intrinsic barrier, which can be calculated as:

(2) $$\Delta G_{0}^{\neq }=\frac{\lambda }{4}$$

In Eq. (2), λ is the reorganisation energy and consists of the inner reorganisation energy of the reactants, $\lambda_{i}$ , and the solvent reorganisation energy, $\lambda_{o}$ . For outer‐sphere electron transfer as in the present case, $\lambda_{i}$ is assumed to be zero (following literature precedents^27^) thus λ is equal to $\lambda_{o}$ .

The reaction energy for the electron transfer step $\Delta G_{r}$ is calculated as the energy of the reaction: Pd(III)‐complex+[Ru(bpy_3_)]^3+^→Pd(IV)‐complex+[Ru(bpy_3_)]^2+^ (i. e. **Int5(SET)** to **Int7**, Scheme 5). The energy for this step is −83.4 kJ mol^−1^.

The reorganisation energy $\lambda =\lambda_{o}$ is calculated using the following equation:^27^, ^28^

(3) $$\lambda_{o}=\frac{N_{A}e^{2}}{4\pi \epsilon_{0}}\left(\frac{1}{\epsilon_{op}}-\frac{1}{\epsilon_{s}}\right)\left(\frac{1}{2r_{1}}+\frac{1}{2r_{2}}-\frac{1}{R}\right)$$

where $N_{A}$ is the Avogadro constant (6.022×10^23^ mol^−1^), *e* is the electronic charge (1.602×10^−19^ C), $\epsilon_{0}$ is the vacuum permittivity (8.854×10^−12^ J^−1^C^2^m^−1^) and, $\epsilon_{op}\;$ and $\epsilon_{s}$ are the optical and static dielectric constant for solvent, respectively. For methanol, $\epsilon_{op}$ is 1.76 and $\epsilon_{s}$ is 32.613. $r_{1}$ , $r_{2}$ and R are the hard sphere radii of the donor, the acceptor, and their sum. In this work, the hard sphere radii approximation of [Ru(bpy)_3_]^3+^ and the Pd(III)‐complex (**Int5(SET)**) were calculated using the VOLUME keyword in Gaussian09. The calculated [Ru(bpy)_3_]^3+^ radius is 6.18 Å and the calculated Pd(III)‐complex radius is 6.47 Å. Using these values in Eq. (3) gives $\lambda_{o}$ =59.1 kJ mol^−1^, and hence $\Delta G_{0}^{\neq }$ =14.8 kJ mol^−1^. Substituting these values for $\Delta G_{0}^{\neq }$ and $\Delta G_{r}$ in Eq. (1), provides a SET barrier, $\Delta G_{ET}^{\neq }$ =2.5 kJ mol^−1^.

## Supporting information

As a service to our authors and readers, this journal provides supporting information supplied by the authors. Such materials are peer reviewed and may be re‐organized for online delivery, but are not copy‐edited or typeset. Technical support issues arising from supporting information (other than missing files) should be addressed to the authors.

Supplementary

## References

[1] T. Cernak , K. D. Dykstra , S. Tyagarajan , P. Vachal , S. W. Krska , Chem. Soc. Rev. 2016, 45, 546–576. 26507237 10.1039/c5cs00628g

[2a] M. A. J. Duncton , MedChemComm 2011, 2, 1135;

[2b] T. Gensch , M. N. Hopkinson , F. Glorius , J. Wencel-Delord , Chem. Soc. Rev. 2016, 45, 2900–2936; 27072661 10.1039/c6cs00075d

[2c] T. A. Bedell , G. A. Hone , D. Valette , J. Q. Yu , H. M. Davies , E. J. Sorensen , Angew. Chem. Int. Ed. Engl. 2016, 55, 8270–8274; 27206223 10.1002/anie.201602024

[2d] A. Sharma , J. F. Hartwig , Nature 2015, 517, 600–604; 25631448 10.1038/nature14127 PMC4311404

[2e] J. J. Topczewski , P. J. Cabrera , N. I. Saper , M. S. Sanford , Nature 2016, 531, 220–224; 26886789 10.1038/nature16957 PMC5082708

[2f] L. McMurray , F. O′Hara , M. J. Gaunt , Chem. Soc. Rev. 2011, 40, 1885–1898; 21390391 10.1039/c1cs15013h

[2g] A. McNally , B. Haffemayer , B. S. Collins , M. J. Gaunt , Nature 2014, 510, 129–133; 24870240 10.1038/nature13389

[2h] F.-L. Zhang , K. Hong , Tuan-Jie Li , H. Park , J.-Q. Yu , Science 2016, 351, 252–256; 26816374 10.1126/science.aad7893 PMC4817545

[2i] F. O′Hara , D. G. Blackmond , P. S. Baran , J. Am. Chem. Soc. 2013, 135, 12122–12134; 23859263 10.1021/ja406223k PMC3776592

[2j] O. Abdulla , A. D. Clayton , R. A. Faulkner , D. M. Gill , C. R. Rice , S. M. Walton , J. B. Sweeney , Chem., Eur. J. 2017, 23, 1494–1497; 27897342 10.1002/chem.201605464

[2k] A. F. Noisier , M. A. Brimble , Chem. Rev. 2014, 114, 8775–8806; 25144592 10.1021/cr500200x

[2l] J. P. Barham , M. P. John , J. A. Murphy , J. Am. Chem. Soc. 2016, 138, 15482–15487; 27809514 10.1021/jacs.6b09690

[2m] J. He , L. G. Hamann , H. M. Davies , R. E. Beckwith , Nat. Commun. 2015, 6, 5943; 25581471 10.1038/ncomms6943

[2n] Q. Michaudel , G. Journot , A. Regueiro-Ren , A. Goswami , Z. Guo , T. P. Tully , L. Zou , R. O. Ramabhadran , K. N. Houk , P. S. Baran , Angew. Chem. Int. Ed. Engl. 2014, 53, 12091–12096; 25244630 10.1002/anie.201407016 PMC4226272

[2o] D. A. Nagib , D. W. MacMillan , Nature 2011, 480, 224–228; 22158245 10.1038/nature10647 PMC3310175

[2p] Y. Zhu , M. Bauer , L. Ackermann , Chem. Eur. J. 2015, 21, 9980–9983. 26037620 10.1002/chem.201501831

[2q] F. Yang , J Koeller , L. Ackermann , Angew. Chem. Int. Ed. 2016, 55, 4759–4762. 10.1002/anie.201512027 26961222

[3a] J. Spencer , B. Z. Chowdhry , A. I. Mallet , R. P. Rathnam , T. Adatia , A. Bashall , F. Rominger , Tetrahedron 2008, 64, 6082–6089;

[3b] R. Khan , R. Felix , P. D. Kemmitt , S. J. Coles , I. J. Day , G. J. Tizzard , J. Spencer , Adv. Synth. Catal. 2016, 358, 98–109. 10.1002/adsc.201700626 PMC6079647 30100832

[4a] E. A. Merritt , B. Olofsson , Angew. Chem. Int. Ed. Engl. 2009, 48, 9052–9070; 19876992 10.1002/anie.200904689

[4b] M. S. Yusubov , A. V. Maskaev , V. V. Zhdankin , Arkivoc 2011, 1, 370–409;

[4c] S. G. Modha , M. F. Greaney , J. Am. Chem. Soc. 2015, 137, 1416–1419. 25588092 10.1021/ja5124754

[5a] D. Kalyani , K. B. McMurtrey , S. R. Neufeldt , M. S. Sanford , J. Am. Chem. Soc. 2011, 133, 18566–18569; 22047138 10.1021/ja208068w PMC3222896

[5b] J. Jiang , W. M. Zhang , J. J. Dai , J. Xu , H. J. Xu , J. Org. Chem. 2017, 82, 3622–3630; 28303717 10.1021/acs.joc.7b00140

[5c] M. Majek , A. Jacobi von Wangelin , Acc. Chem. Res. 2016, 49, 2316–2327; 27669097 10.1021/acs.accounts.6b00293

[5d] L. Marzo , I. Ghosh , F. Esteban , B. König , ACS Catal. 2016, 6780–6784;

[5e] D. P. Hari , P. Schroll , B. Konig , J. Am. Chem. Soc. 2012, 134, 2958–2961. 22296099 10.1021/ja212099r

[6] S. J. Coles , P. A. Gale , Chem. Sci. 2012, 3, 683–689.

[7a] J. F. Bunnett , R. E. Zahler , Chem. Rev. 1951, 273–412;

[7b] I. K. Barbem , H. Suschitz , J. Chem. Soc. 1960, 2735–2739.

[8] J. R. Atack , K. A. Wafford , S. J. Tye , S. M. Cook , B. Sohal , A. Pike , C. Sur , D. Melillo , L. Bristow , F. Bromidge , I. Ragan , J. Kerby , L. Street , R. Carling , J. L. Castro , P. Whiting , G. R. Dawson , R. M. McKernan , J. Pharmacol. Exp. Ther. 2006, 316, 410–422. 16183706 10.1124/jpet.105.089920

[9] D. L. Davies , S. M. A. Donald , S. A. Macgregor , J. Am. Chem. Soc. 2005, 127, 13754–11375. 16201772 10.1021/ja052047w

[10] A. Vigalok , O. Uzan , L. J. W. Shimon , Y. Ben-David , J. M. L. Martin , D. Milstein , J. Am. Chem. Soc. 1998, 120, 12539–12544.

[11] F. Teplý , Collection Czech Chem. Comm. 2011, 76, 859–917.

[12a] J. R. Khusnutdinova , N. P. Rath , L. M. Mirica , J. Am. Chem. Soc. 2010, 132, 7303–7305. 20462195 10.1021/ja103001g

[12b] D. C. Powers , T. Ritter , Nat. Chem. 2009, 1, 302–309 21500602 10.1038/nchem.246

[13a] R. A. Marcus , Ann. Rev. Phys. Chem. 1964, 15, 155–196;

[13b] R. A. Marcus , J. Chem. Phys. 1956, 24, 966–978;

[13c] R. A. Marcus , J. Chem. Phys. 1956, 24, 979–989;

[13d] J.-M. Saveant , J. Am. Chem. Soc. 1987, 109, 6788–6795.

[14] Q. Zhang , Z.-Q. Zhang , Y. Fu , H.-Z. Yu , ACS Catal. 2016, 6, 798–808.

[15] A. Ariafard , C. J. T. Hyland , A. J. Canty , M. Sharma , B. F. Yates , Inorg. Chem. 2011, 50, 6449–6457. 21671562 10.1021/ic102323s

[16] A. J. Canty , A. Ariafard , M. S. Sanford , B. F. Yates , Organometallics 2013, 32, 544−555.

[17] H. Ossor , M. Pfeffer , J. T. B. H. Jastrzebski , C. H. Stam , Inorg. Chem. 1987, 26, 1169–1117 1161.

[18a] P. Hanson , J. R. Jones , A. B. Taylor , P. H. Walton , A. W. Timms , J. Chem. Soc. Perkin Trans. 2 2002, 1135–1150;

[18b] B. Schmidt , R. Berger , F. Holter , Org. Biomol. Chem. 2010, 8, 1406–1414. 20204215 10.1039/b924619c

[19] S. H. Korzeniowski , A. Leopold , J. R. Beadle , M. F. Ahern , W. A. Sheppard , R. K. Khanna , G. W. Gokel , J. Org. Chem. 1981, 46, 2153–2159.

[20] M. J. Frisch, G. W. Trucks, H. B. Schlegel, G. E. Scuseria, M. A. Robb, et al., Gaussian Inc., Wallingford, CT, **2009**.

[21] D. Andrae , U. HuBermann , M. Dolg , H. Stoll , H. PreuB , Theor. Chim. Acta 1990, 77, 123–141.

[22] J. P. Perdew , K. Burke , M. Ernzerhof , Phys. Rev. Lett. 1997, 77, 1396. 10.1103/PhysRevLett.77.3865 10062328

[23a] S. Boonseng , G. W. Roffe , J. Spencer , H. Cox , Dalton Trans. 2015, 44, 7570–7577; 25811120 10.1039/c5dt00031a

[23b] P. Surawatanawong , M. B. Hall , Organometallics 2008, 27, 6222–6232.

[24] J. D. Chai , M. Head-Gordon , Phys. Chem. Chem. Phys. 2008, 10, 6615–6620. 18989472 10.1039/b810189b

[25a] Y. Minenkov , G. Occhipinti , V. R. Jensen , J. Phys. Chem. A 2009, 113, 11833–11844; 19736907 10.1021/jp902940c

[25b] N. Sieffert , M. Buhl , Inorg. Chem. 2009, 48, 4622–4624; 19382761 10.1021/ic900347e

[25c] M. L. Laury , A. K. Wilson , J. Chem. Theory Comput. 2013, 9, 3939–3946; 26592389 10.1021/ct400379z

[25d] Y. Zhao , D. G. Truhlar , J. Chem. Theory Comput. 2011, 7, 669–676.

[26a] V. Barone , M. Cossi , J. Phys. Chem. A 1998, 102, 1995–2001;

[26b] M. Cossi , N. Rega , G. Scalmani , V. Barone , J. Comp. Chem. 2003, 24, 669–681. 12666158 10.1002/jcc.10189

[27a] G. O. Jones , P. Liu , K. N. Houk , S. L. Buchwald , J. Am. Chem. Soc. 2010, 132, 6205–6213; 20387898 10.1021/ja100739h PMC2908497

[27b] C. Y. Lin, M. L. Coote, A. Gennaro, K. Matyjaszewski, *J. Am. Chem. Soc*, **2008**, *130*, 12762–12774. 10.1021/ja8038823 18761460

[28] R. A. Marcus , N. Sutin , Biochimica et Biophysica Acta 1985, 811, 265–322.

