# Supplementary material for: Combining Sanford Arylations on Benzodiazepines with the Nuisance Effect
Source: Adv Synth Catal. 2017 Aug 2;359(18):3261–9. doi: 10.1002/adsc.201700626 (PMC6079647; doi:10.1002/adsc.201700626)
Supplement: Supplementary file 1 — Supplementary [file ADSC-359-3261-s001.pdf]

Advanced Synthesis & Catalysis

Supporting Information

Combining Sanford Arylations with the *Nuisance Effect*

Raysa Khan,[a] Sarote Boonseng,[a] Paul D. Kemmitt,[b] Robert Felix,[c] Simon J. Coles,[d] Graham J. Tizzard,[d] Gareth Williams,[e] Olivia Simmonds,[e] Jessica-Lily Harvey,[e] John Atack,[e] Hazel Cox [a] and John Spencer[a,*]

[a] Department of Chemistry, School of Life Sciences, University of Sussex, Falmer, BN1 9QJ, UK. Email: j.spencer@sussex.ac.uk.

[b] AstraZeneca, Mereside Alderley Park, Macclesfield, SK10 4TG, UK.

[c] Tocris Bioscience, Tocris House, IO Centre, Moorend Farm Avenue, Bristol, BS11 0QL, UK.

[d] UK National Crystallography Service, School of Chemistry, University of Southampton, Highfield, Southampton, SO17 1BJ, UK.

[e] Sussex Drug Discovery Centre, School of Life Sciences, University of Sussex, Falmer, BN1 9QJ, UK.

Synthetic Chemistry

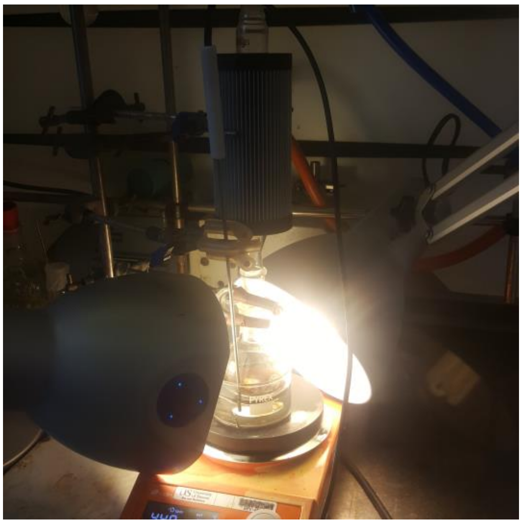

Fig. 1: Reaction using (26 W) lightbulbs

Spectral Data

^{1}H NMR of all compounds; ^{1}H and ^{13}C NMR spectra of the novel compounds.

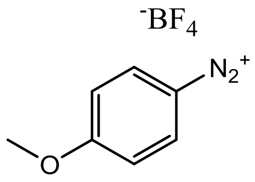

2d

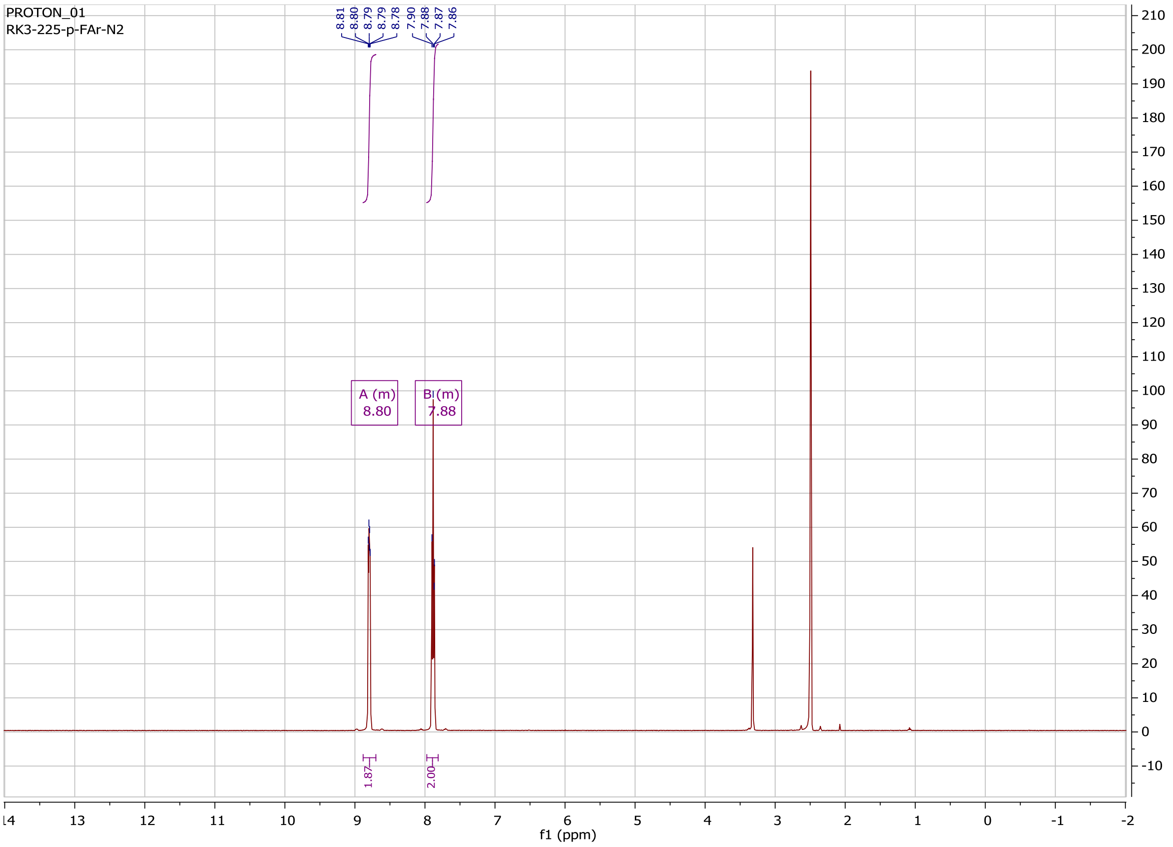

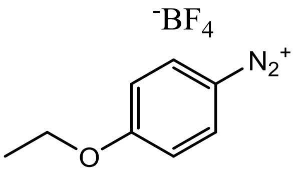
-BF4
N2+
O

2e

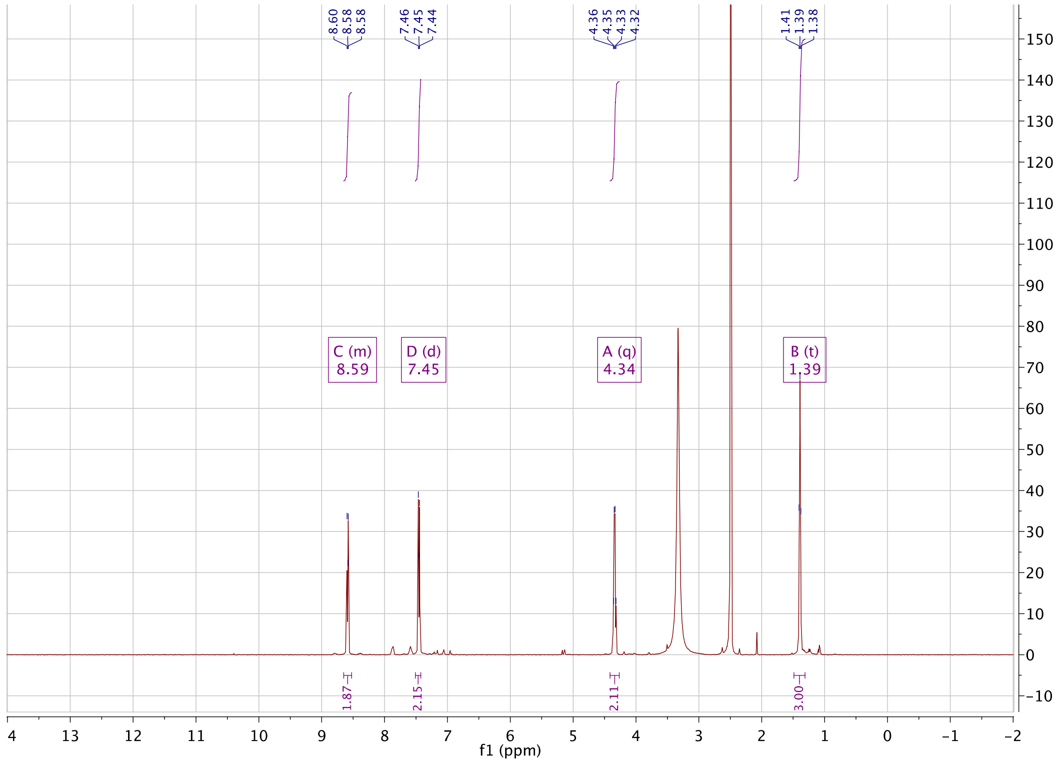
8.60
8.58
8.58
7.46
7.45
7.44
4.36
4.35
4.33
4.32
1.41
1.39
1.38
C (m)
8.59
D (d)
7.45
A (q)
4.34
B (t)
1.39
1.87
2.15
2.11
3.00
f1 (ppm)

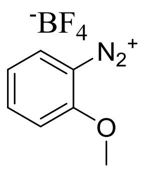

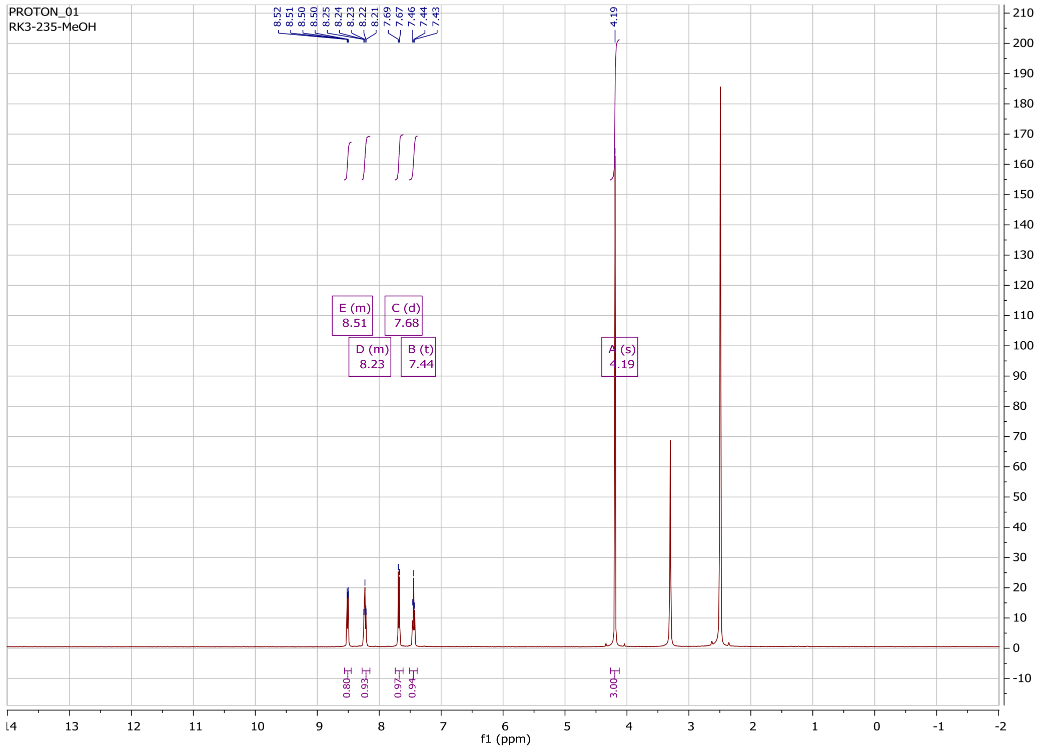

PROTON_01
RK3-235-MeOH
E (m) 8.51
D (m) 8.23
C (d) 7.68
B (t) 7.44
A (s) 4.19
f1 (ppm)

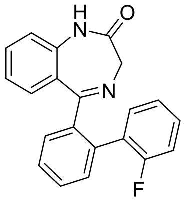
H
N
O
N
F

3a

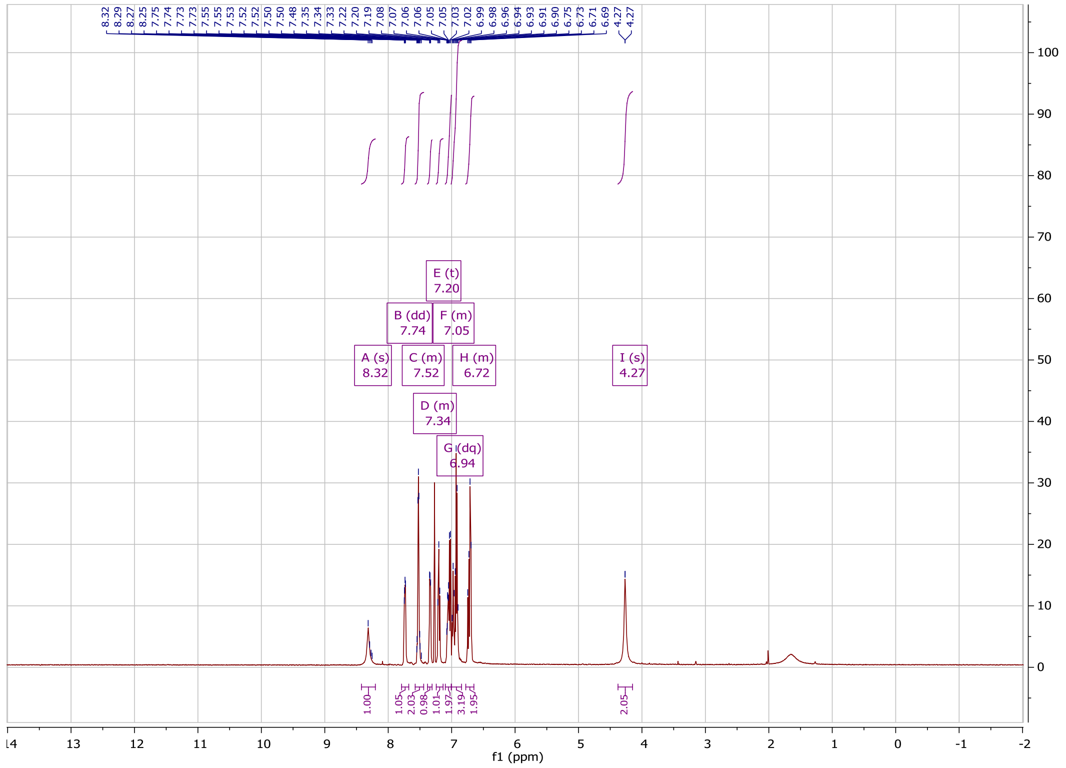
A (s)
8.32
B (dd)
7.74
C (m)
7.52
D (m)
7.34
E (t)
7.20
F (m)
7.05
G (dq)
6.94
H (m)
6.72
I (s)
4.27
1.00
1.05
2.03
0.98
1.01
1.97
3.19
1.95
2.05
f1 (ppm)

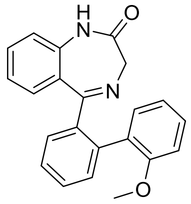

4a

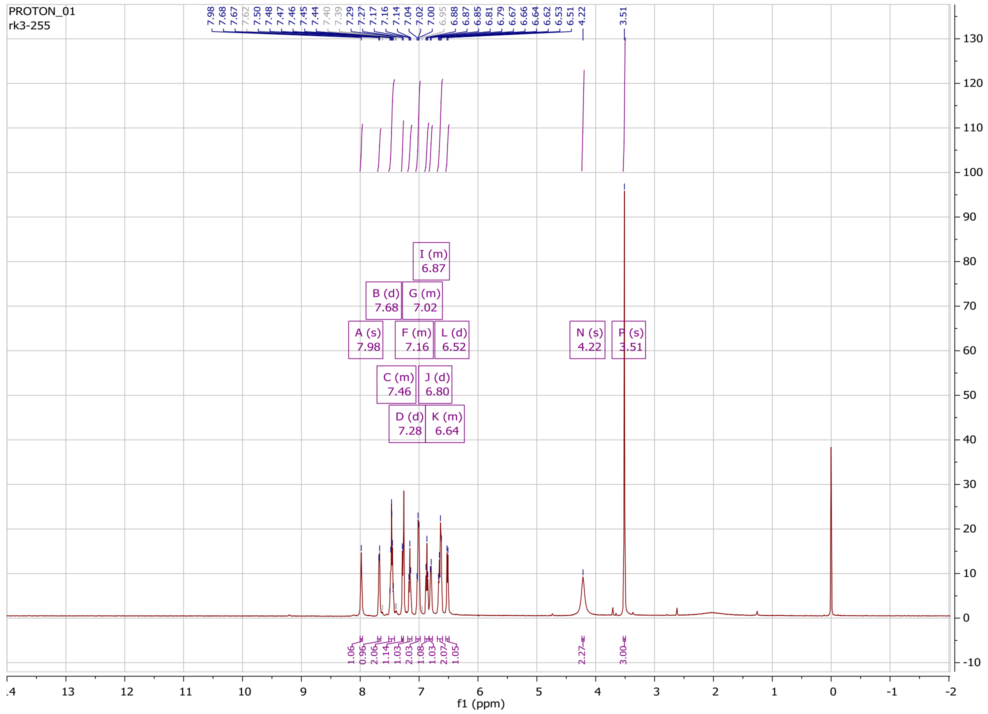

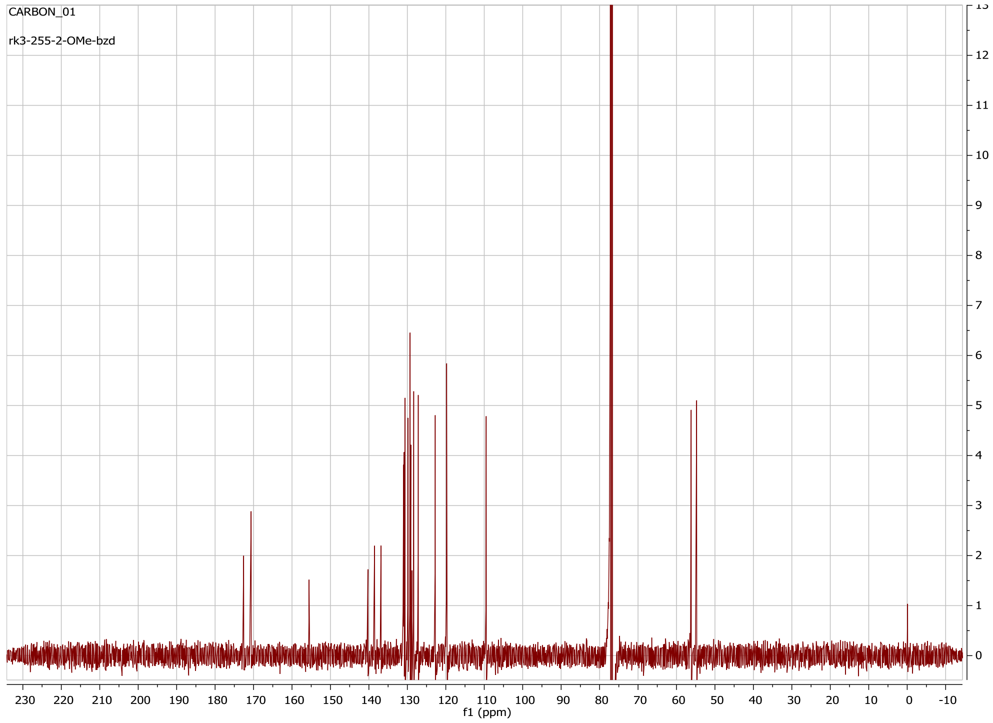

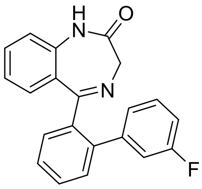
H
N
O
N
F

3b

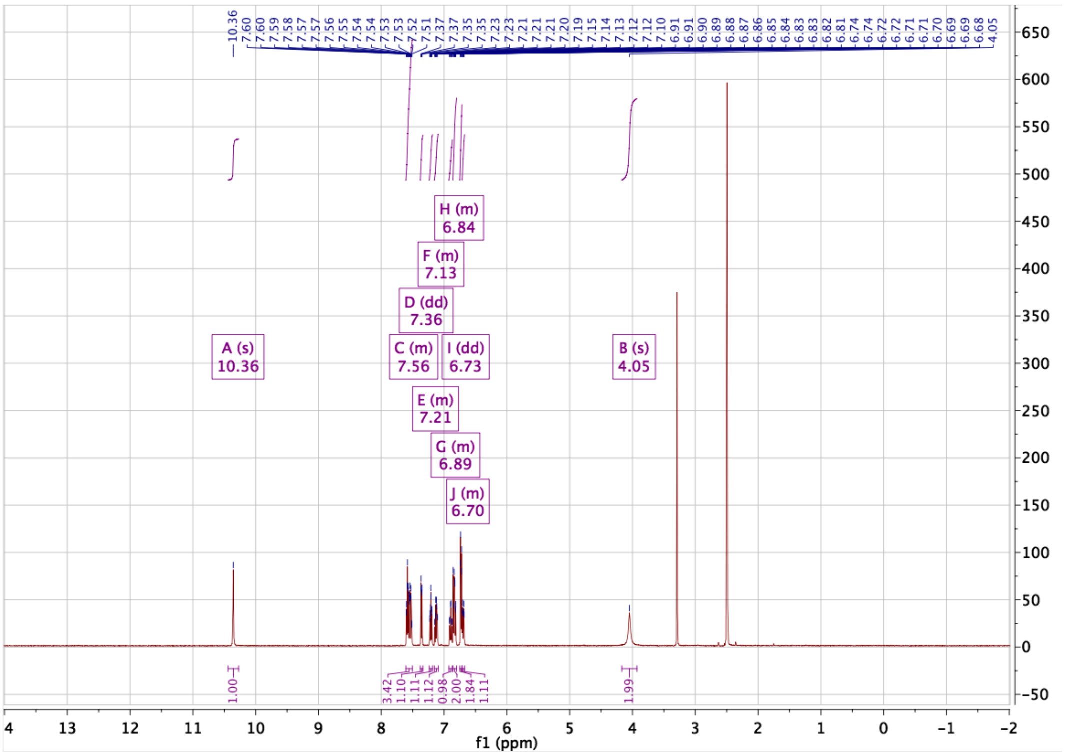
A (s)
10.36
B (s)
4.05
C (m)
7.56
D (dd)
7.36
E (m)
7.21
F (m)
7.13
G (m)
6.89
H (m)
6.84
I (dd)
6.73
J (m)
6.70
1.00
3.42
1.10
1.11
1.12
0.98
2.00
1.84
1.11
1.99
f1 (ppm)

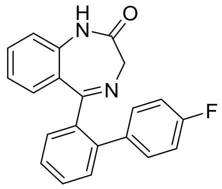

3c

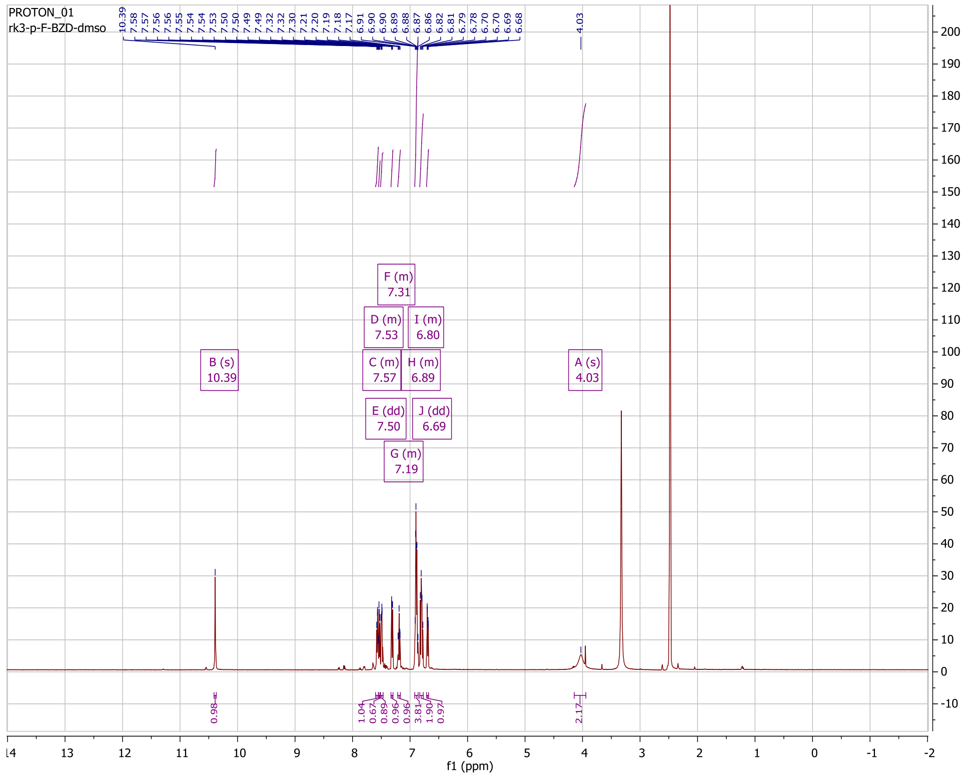
PROTON_01
rk3-p-F-BZD-dmso
F (m) 7.31
D (m) 7.53
I (m) 6.80
B (s) 10.39
C (m) 7.57
H (m) 6.89
A (s) 4.03
E (dd) 7.50
J (dd) 6.69
G (m) 7.19
f1 (ppm)

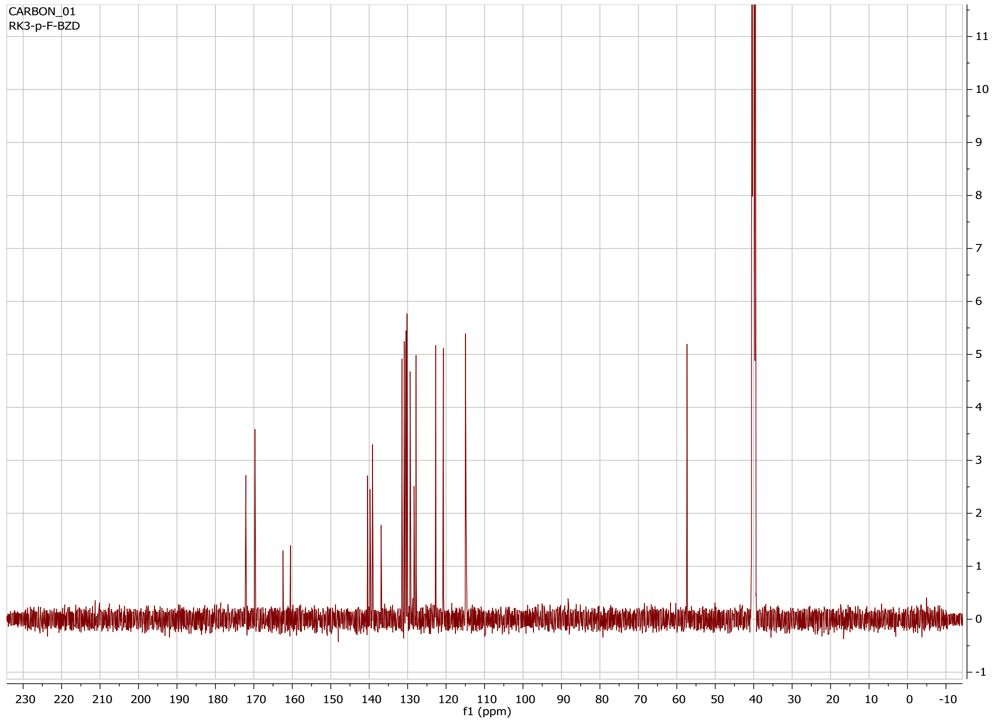
CARBON_01
RK3-p-F-BZD
f1 (ppm)

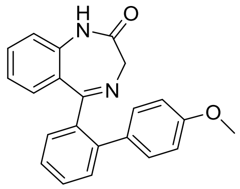

4c

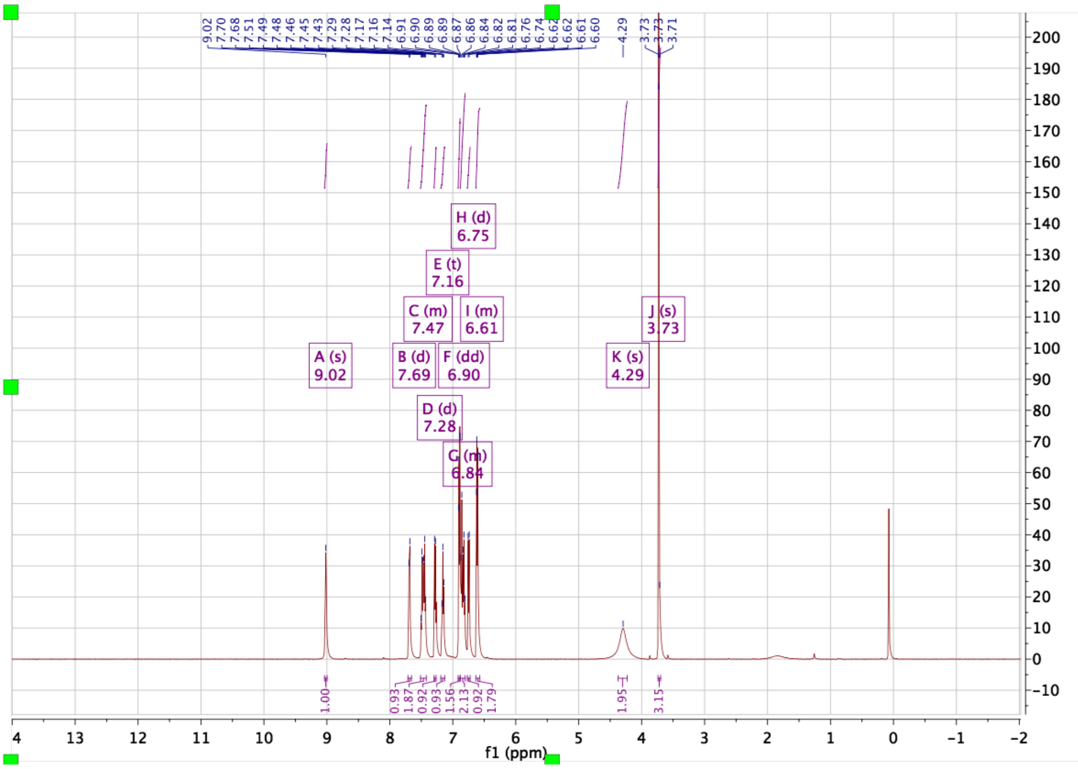

9.02 7.70 7.68 7.51 7.49 7.48 7.46 7.45 7.43 7.29 7.28 7.17 7.16 7.14 6.91 6.90 6.89 6.89 6.87 6.86 6.84 6.82 6.81 6.76 6.74 6.62 6.62 6.61 6.60
4.29
3.73 3.73 3.71
H (d) 6.75
E (t) 7.16
C (m) 7.47
I (m) 6.61
J (s) 3.73
A (s) 9.02
B (d) 7.69
F (dd) 6.90
K (s) 4.29
D (d) 7.28
G (m) 6.84
1.00
0.93 1.87 0.92 0.93 1.56 2.13 0.92 1.79
1.95
3.15
f1 (ppm)

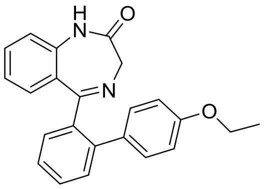

4d

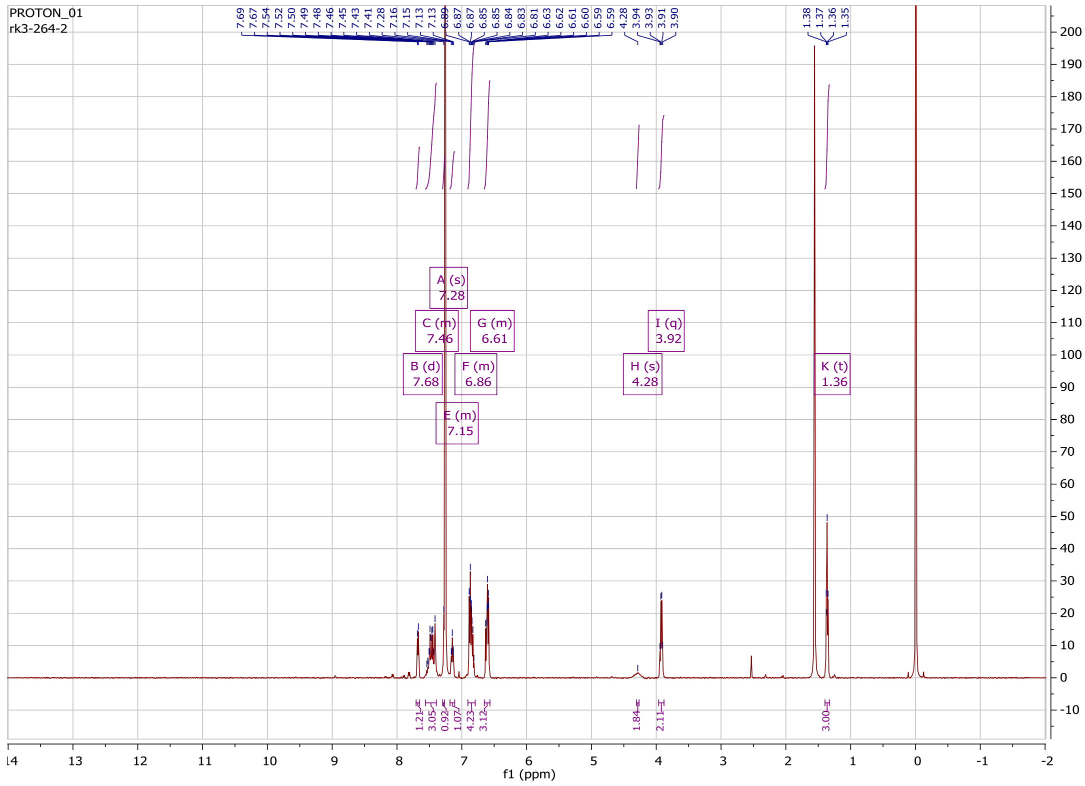
PROTON_01
rk3-264-2
A (s)
7.28
B (d)
7.68
C (m)
7.46
E (m)
7.15
F (m)
6.86
G (m)
6.61
H (s)
4.28
I (q)
3.92
K (t)
1.36
f1 (ppm)

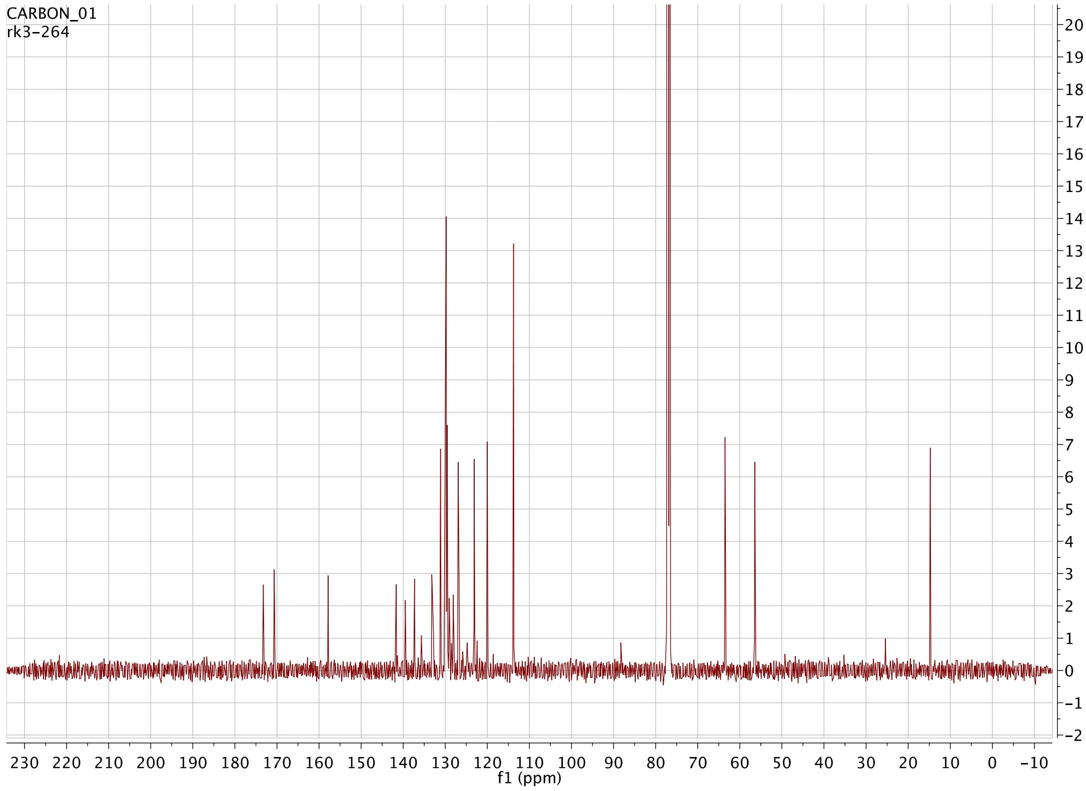
CARBON_01
rk3-264
f1 (ppm)

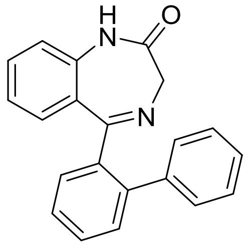

4e

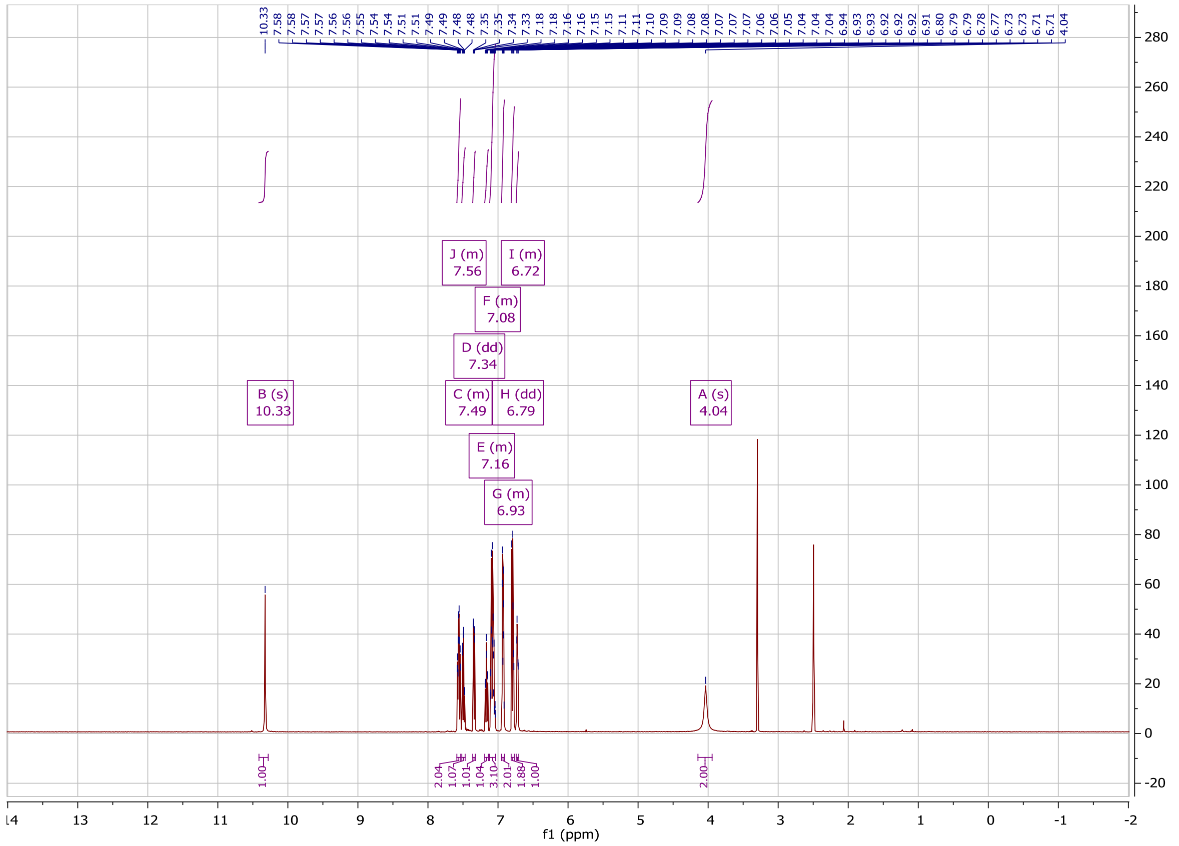

10.33
7.58
7.58
7.57
7.57
7.56
7.56
7.55
7.54
7.54
7.51
7.51
7.49
7.49
7.48
7.48
7.35
7.35
7.34
7.33
7.18
7.18
7.16
7.16
7.15
7.15
7.11
7.11
7.10
7.09
7.09
7.08
7.08
7.07
7.07
7.07
7.06
7.06
7.05
7.04
7.04
7.04
6.94
6.93
6.93
6.92
6.92
6.92
6.91
6.80
6.79
6.79
6.78
6.77
6.73
6.73
6.71
6.71
4.04
J (m)
7.56
I (m)
6.72
F (m)
7.08
D (dd)
7.34
B (s)
10.33
C (m)
7.49
H (dd)
6.79
A (s)
4.04
E (m)
7.16
G (m)
6.93
1.00
2.04
1.07
1.01
1.04
3.10
2.01
1.88
1.00
2.00
f1 (ppm)

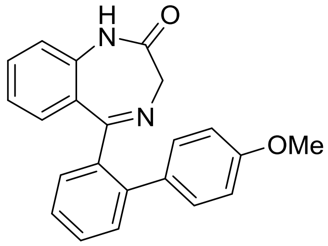
H
N
O
N
OMe

4f

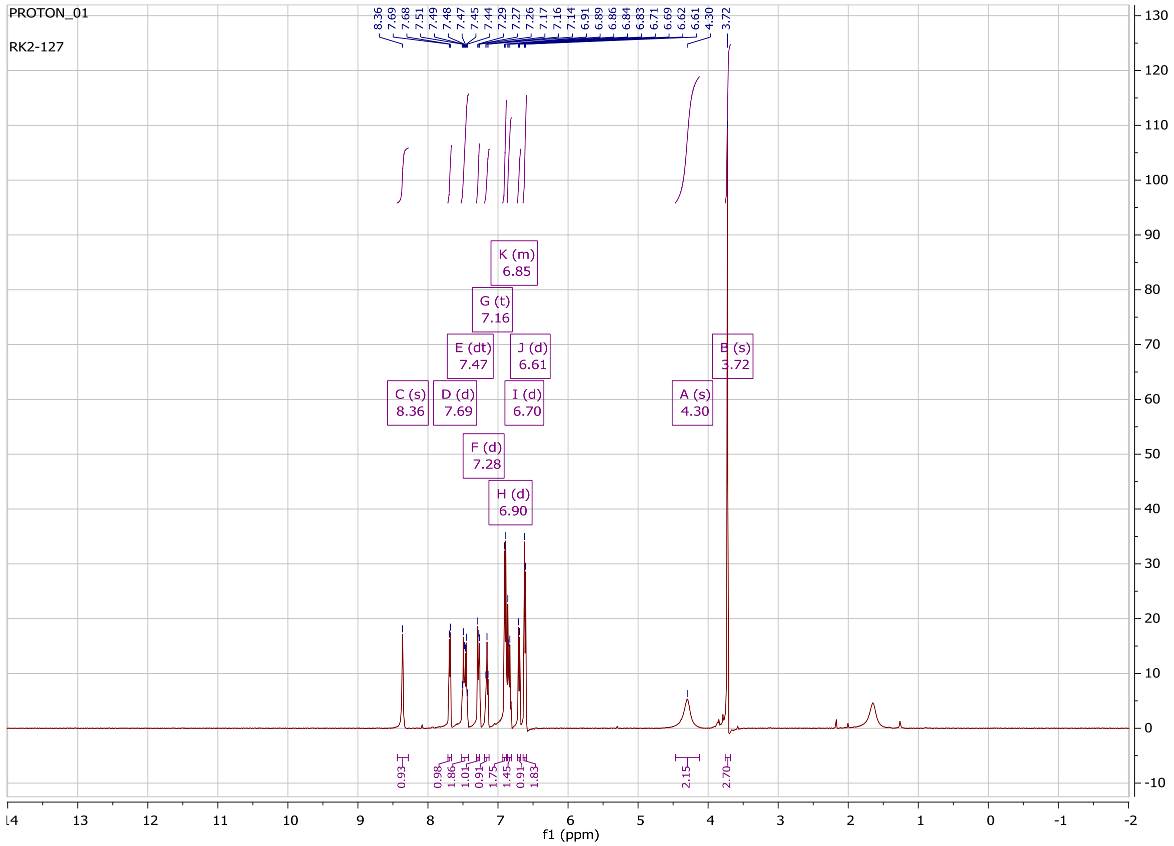
PROTON_01
RK2-127
8.36
7.69
7.68
7.51
7.49
7.48
7.47
7.45
7.44
7.29
7.27
7.26
7.17
7.16
7.14
6.91
6.89
6.86
6.84
6.83
6.71
6.69
6.62
6.61
4.30
3.72
K (m) 6.85
G (t) 7.16
E (dt) 7.47
J (d) 6.61
B (s) 3.72
C (s) 8.36
D (d) 7.69
I (d) 6.70
A (s) 4.30
F (d) 7.28
H (d) 6.90
0.93
0.98
1.86
1.01
0.91
1.75
1.45
0.91
1.83
2.15
2.70
f1 (ppm)

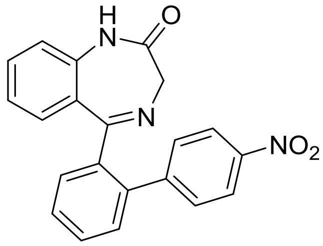

4g

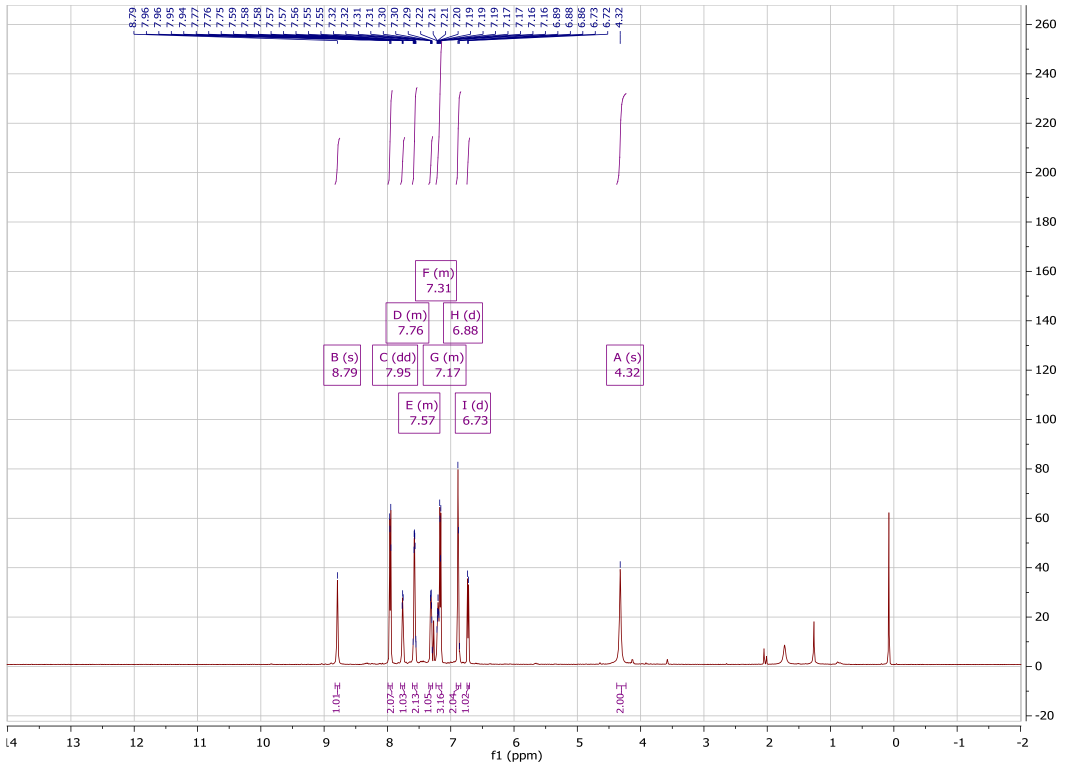
B (s) 8.79
C (dd) 7.95
D (m) 7.76
E (m) 7.57
F (m) 7.31
G (m) 7.17
H (d) 6.88
I (d) 6.73
A (s) 4.32
f1 (ppm)

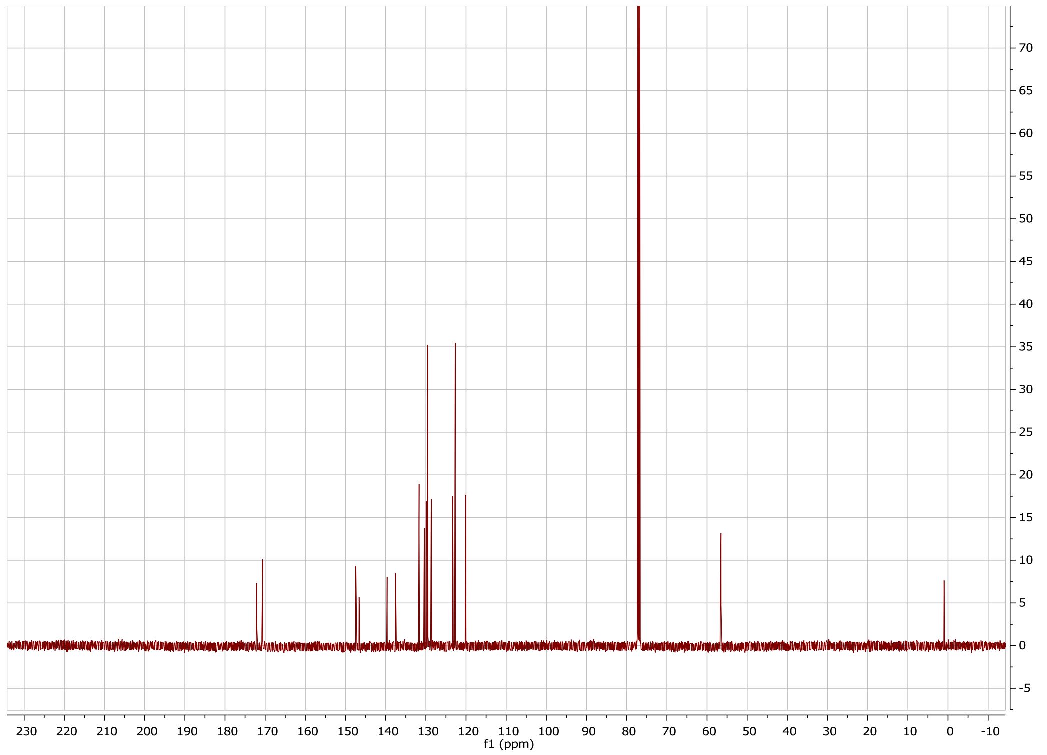
f1 (ppm)

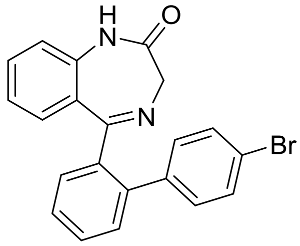

4h

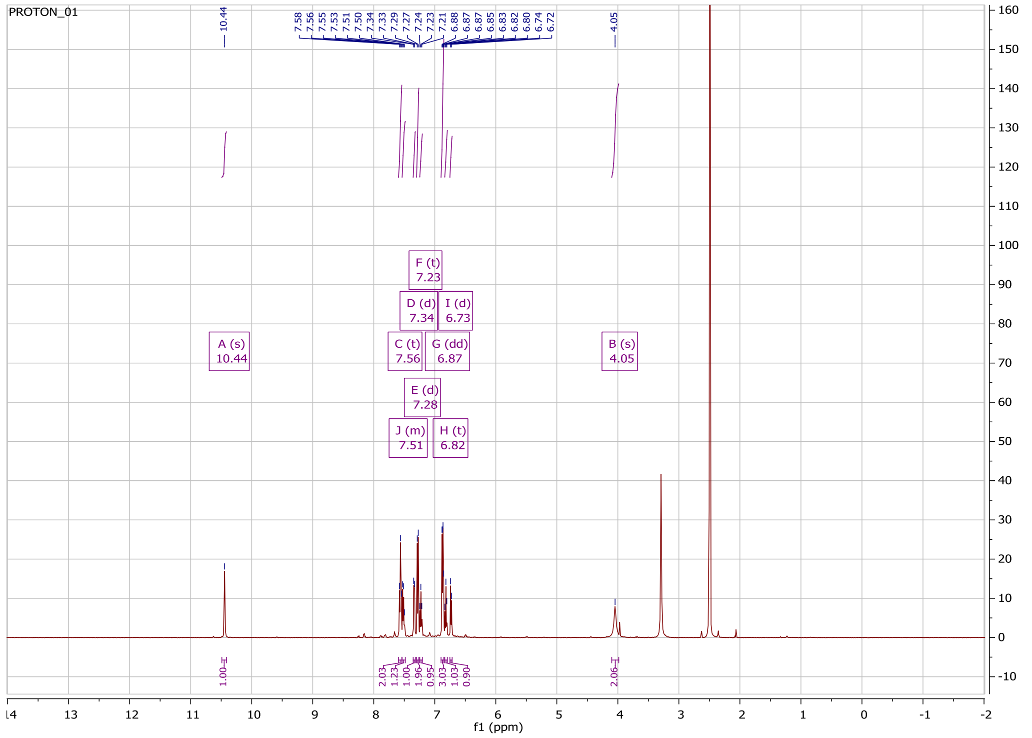
PROTON_01
A (s)
10.44
B (s)
4.05
C (t)
7.56
D (d)
7.34
E (d)
7.28
F (t)
7.23
G (dd)
6.87
H (t)
6.82
I (d)
6.73
J (m)
7.51
f1 (ppm)

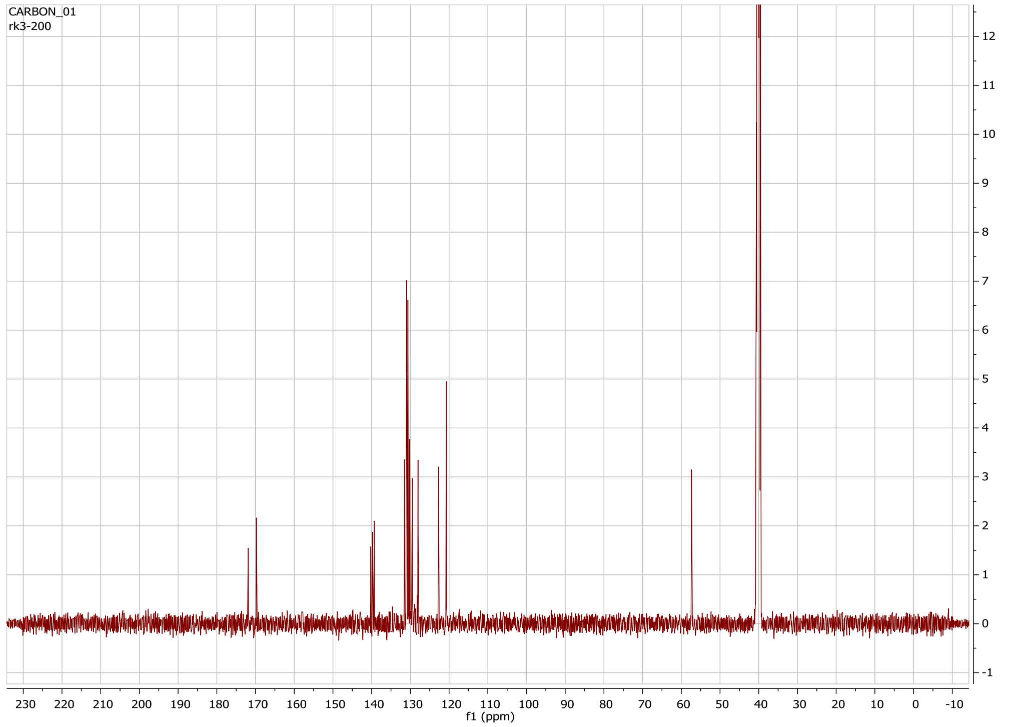
CARBON_01
rk3-200
f1 (ppm)

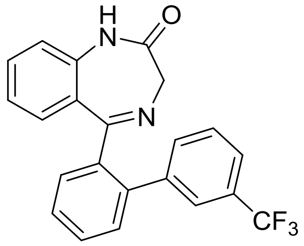

4i

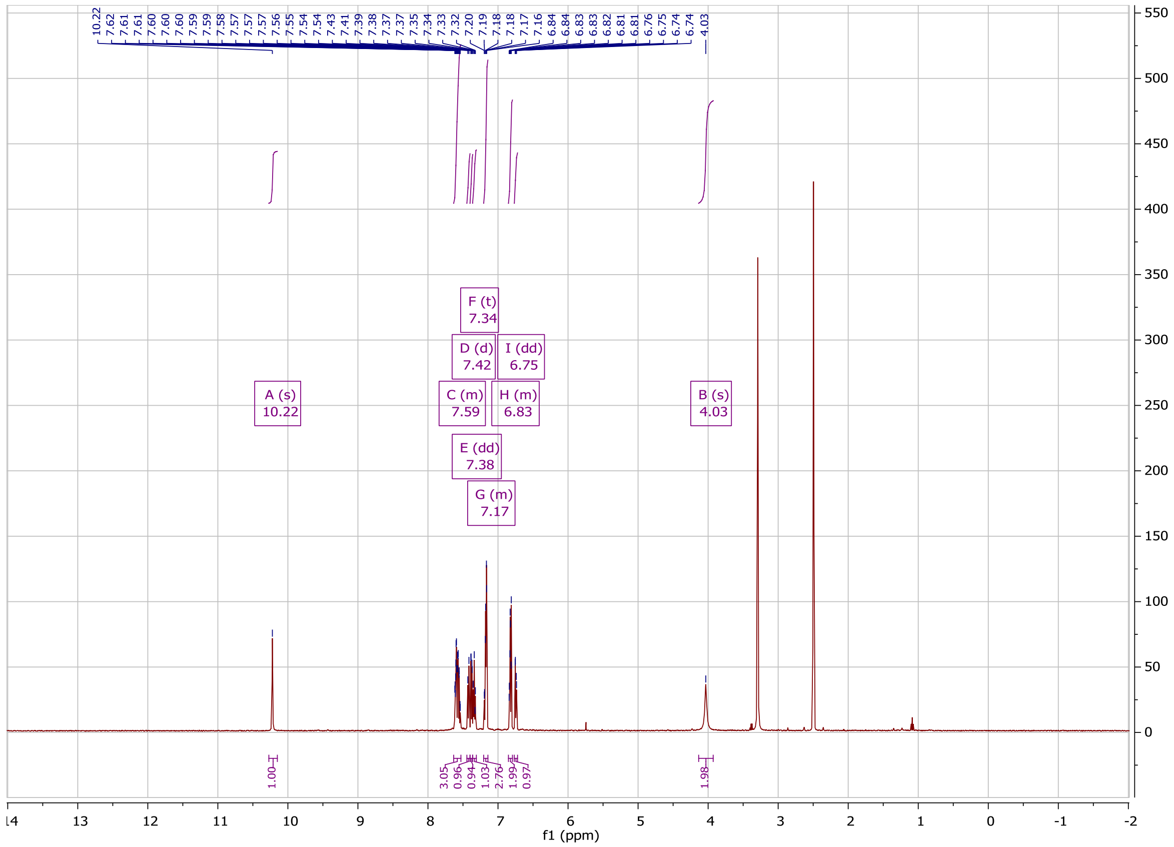
A (s)
10.22
B (s)
4.03
C (m)
7.59
D (d)
7.42
E (dd)
7.38
F (t)
7.34
G (m)
7.17
H (m)
6.83
I (dd)
6.75
f1 (ppm)

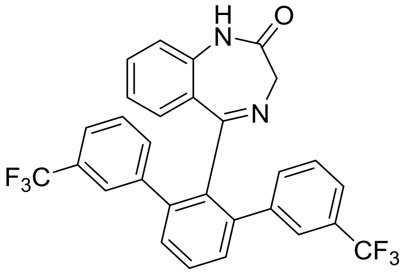
H
N
O
N
F3C
CF3

4i’

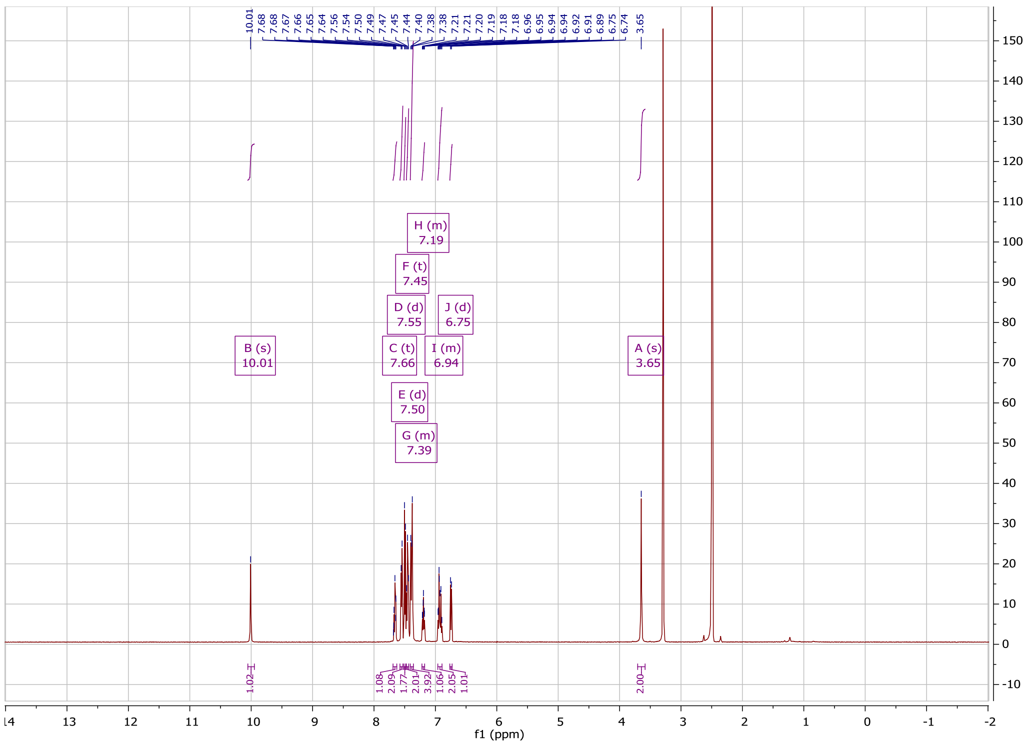
10.01
3.65
B (s) 10.01
C (t) 7.66
D (d) 7.55
E (d) 7.50
F (t) 7.45
G (m) 7.39
H (m) 7.19
I (m) 6.94
J (d) 6.75
A (s) 3.65
1.02
2.00
f1 (ppm)

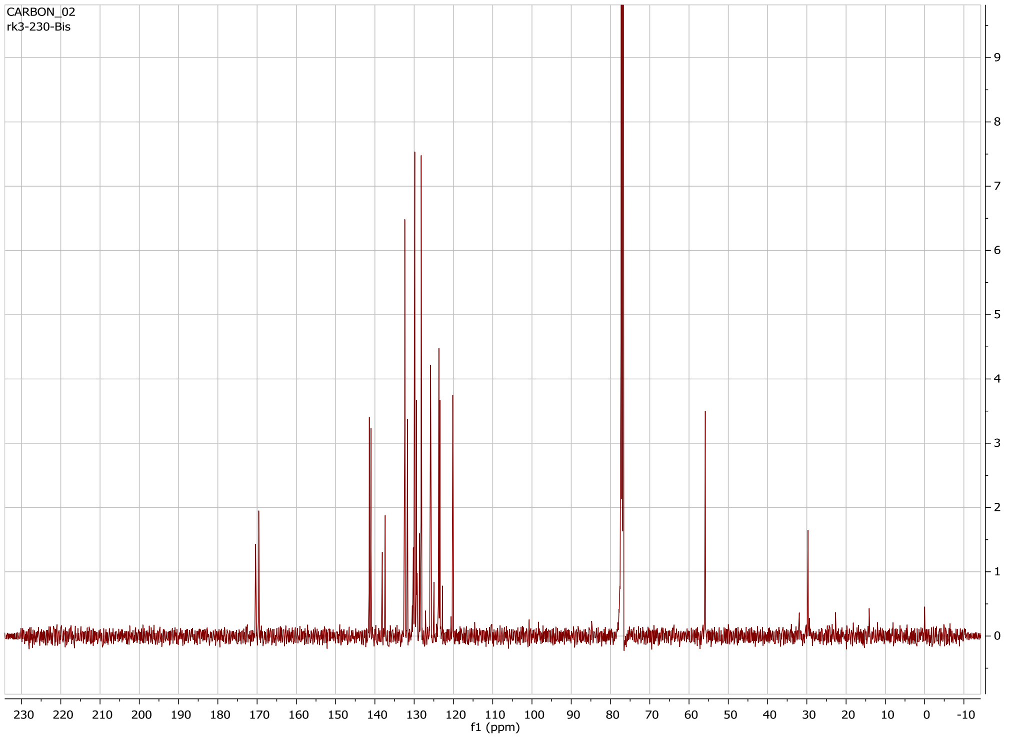
CARBON_02
rk3-230-Bis
f1 (ppm)

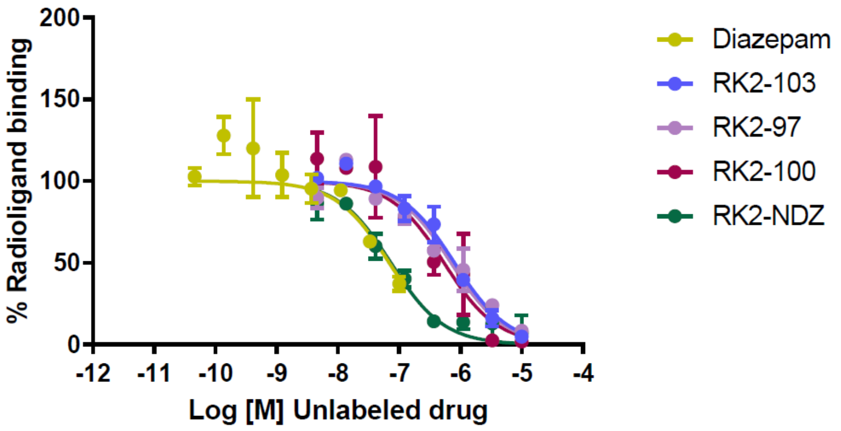

Diazepam IC50= 68.77nM

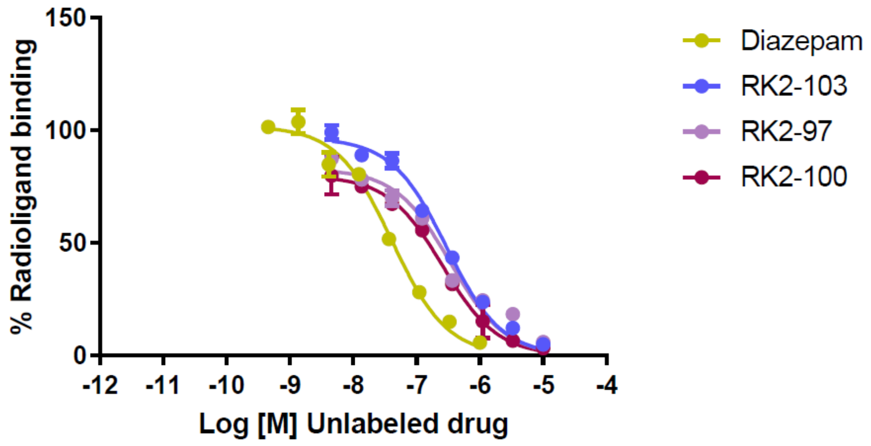

Diazepam IC50 = 41.15nM

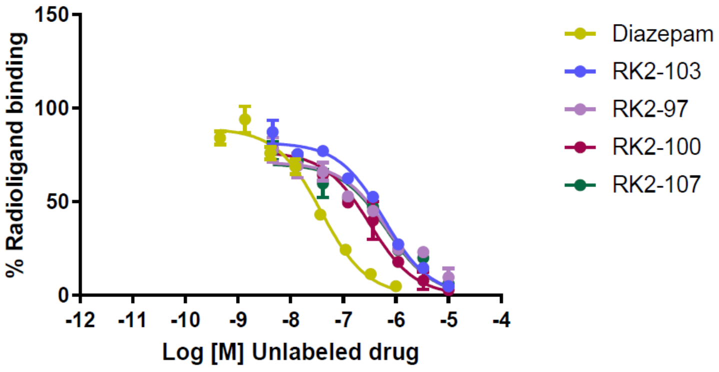

Diazepam IC50= 38.53 nM

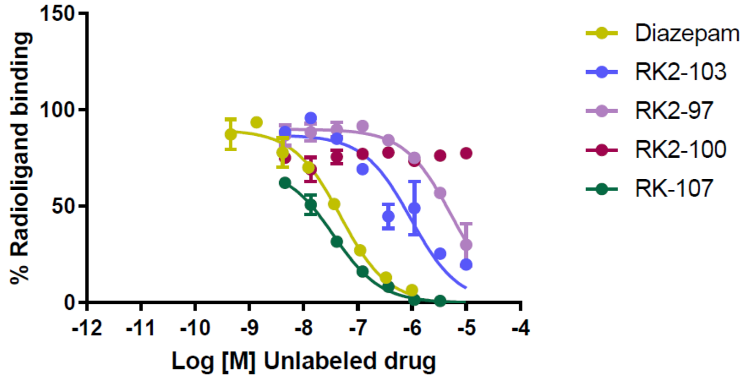

Diazepam IC50 = 48.42nM

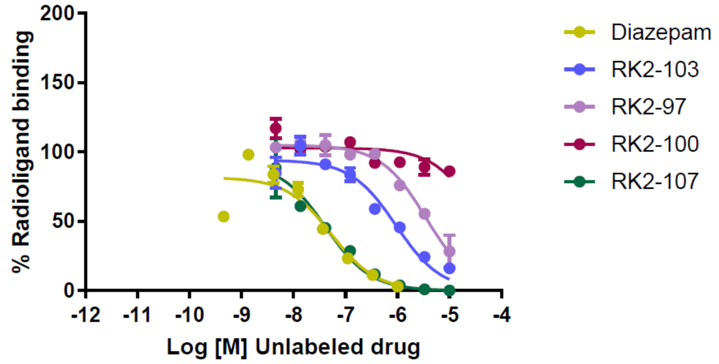

Diazepam IC50= 53.53 nM
